# Comparisons of Isolation Methods, Structural Features, and Bioactivities of the Polysaccharides from Three Common Panax Species: A Review of Recent Progress

**DOI:** 10.3390/molecules26164997

**Published:** 2021-08-18

**Authors:** Hongyu Qi, Zepeng Zhang, Jiaqi Liu, Zhaoqiang Chen, Qingxia Huang, Jing Li, Jinjin Chen, Mingxing Wang, Daqing Zhao, Zeyu Wang, Xiangyan Li

**Affiliations:** 1 Jilin Ginseng Academy, Key Laboratory of Active Substances and Biological Mechanisms of Ginseng Efficacy, Ministry of Education, Jilin Provincial Key Laboratory of Bio-Macromolecules of Chinese Medicine, Changchun University of Chinese Medicine, Changchun 130117, China; qhy17843303123@163.com (H.Q.); ljqly567@163.com (J.L.); chen030814@163.com (Z.C.); hqx19890928@163.com (Q.H.); 15703416591@163.com (J.L.); chen1091073165@163.com (J.C.); zhaodaqing1963@163.com (D.Z.); 2 Research Center of Traditional Chinese Medicine, College of Traditional Chinese Medicine, Changchun University of Chinese Medicine, Changchun 130021, China; zzp9762@126.com (Z.Z.); cc_wmx@163.com (M.W.); 3 College of Acupuncture and Tuina, Changchun University of Chinese Medicine, Changchun 130117, China; 4 Department of Scientific Research, Changchun University of Chinese Medicine, Changchun 130117, China

**Keywords:** *Araliaceae* family, polysaccharides, extraction, purification, structural features, pharmacological functions

## Abstract

*Panax* spp. (*Araliaceae* family) are widely used medicinal plants and they mainly include *Panax ginseng* C.A. Meyer*, Panax quinquefolium* L. (American ginseng)*,* and *Panax notoginseng* (notoginseng). Polysaccharides are the main active ingredients in these plants and have demonstrated diverse pharmacological functions, but comparisons of isolation methods, structural features, and bioactivities of these polysaccharides have not yet been reported. This review summarizes recent advances associated with 112 polysaccharides from ginseng, 25 polysaccharides from American ginseng, and 36 polysaccharides from notoginseng and it compares the differences in extraction, purification, structural features, and bioactivities. Most studies focus on ginseng polysaccharides and comparisons are typically made with the polysaccharides from American ginseng and notoginseng. For the extraction, purification, and structural analysis, the processes are similar for the polysaccharides from the three Panax species. Previous studies determined that 55 polysaccharides from ginseng, 18 polysaccharides from American ginseng, and 9 polysaccharides from notoginseng exhibited anti-tumor activity, immunoregulatory effects, anti-oxidant activity, and other pharmacological functions, which are mediated by multiple signaling pathways, including mitogen-activated protein kinase, nuclear factor kappa B, or redox balance pathways. This review can provide new insights into the similarities and differences among the polysaccharides from the three Panax species, which can facilitate and guide further studies to explore the medicinal properties of the *Araliaceae* family used in traditional Chinese medicine.

## 1. Introduction

Ginseng (*Panax* spp., *Araliaceae* family*)* is a medical and edible herb that has traditionally been used for thousands of years to regulate bodily functions and exert multiple protective effects [1,2]. Currently, representative Panax members include *Panax ginseng* C.A. Meyer (Asian or Korean ginseng, Renshen), *Panax quinquefolium* L. (American ginseng), and *Panax notoginseng* (Burk) F. H. Chen (notoginseng), which are of importance for their use in medicine and as dietary supplements for the prevention and treatment of cardiovascular diseases, neurodegenerative diseases, cancer, fatigue, aging, and metabolic diseases [3,4,5]. At present, approximately 200 active substances in these three species have been discovered, including saponins [6,7,8], polysaccharides [9,10], oligosaccharides [11,12], polyacetylenes [13,14], peptides [15,16], and bioactive proteins [17]. Polysaccharides are the main active components and have demonstrated diverse pharmacological functions, such as anti-tumor [18,19], immune system modulation [20,21,22,23], anti-oxidant [24,25] and anti-aging [26] activities, as well as others [27,28].

Currently, most studies focus on the purification, structural analysis, and bioactivities of polysaccharides from the three above-mentioned different Panax species [29,30,31]. Ginseng polysaccharides have been deeply studied and recent findings have been extensively summarized [9,10]. Recently, the polysaccharides from two other species were examined, and most studies focus on the structural features and immune-stimulating effects of polysaccharides isolated from American ginseng and notoginseng [32,33]. The similarities and differences between these polysaccharides from Panax species and their effects on isolation and purification, structural characteristics, and biological functions remain unclear and have been not summarized. In this review, we summarize the methods of extraction and purification, structural characteristics, and the main biological activities of polysaccharides from three common ginseng species, which will provide new insights into the understanding of current research and future direction for these polysaccharides.

## 2. Extraction and Purification Methods

### 2.1. Extraction and Precipitation

To obtain polysaccharide fractions from the various Panax species, different extraction methods are used to extract different polysaccharides, including hot water/ethanol extraction [29,34,35,36], alkaline extraction [37,38], enzymatic extraction [39,40,41], and ethylenediaminetetraacetic acid (EDTA) extraction [42]. Other new methods, such as ultrasonic extraction [43,44] and microwave extraction [45], have been performed to extract polysaccharides from Panax species and these can improve extraction efficiency and decrease extraction time.

Most studies report that hot water extraction was used to isolate the polysaccharides from three Panax species, which is the most classical and convenient method for isolating water-soluble crude polysaccharide [29,46]. Briefly, the roots of Panax species were soaked in water overnight and boiled in water (2–4 times, 2–6 h each time) to obtain the supernatants, which were centrifuged at 5000 rpm for 30 min and concentrated under vacuum. The crude extracts were precipitated with cold ethanol at 4 °C for 24 h and deproteinated by the Sevag method to obtain crude polysaccharides [9]. Some reports showed that 80–95% ethanol was used to remove lipophilic compounds and increase purity before water extraction [36,47], which can increase the rate of extraction of polysaccharides from ginseng [48]. In fact, different extraction methods have been used to prevent the destruction of polysaccharide structures and bioactivities. EDTA extraction was used to extract pectin-type polysaccharides with high efficiency [42].

After water extraction, alkali extraction of acid polysaccharides with Na_2_CO_3_, 1 M KOH, or 4 M KOH is suitable when high temperature may degrade the activities of some polysaccharides [37,49]. Furthermore, enzyme-assisted extraction methods can destroy granular starch and enable a higher extraction yield of polysaccharides. Two enzymes, α-amylase and cellulase, were used to effectively extract ginseng polysaccharides with different structures and activities, compared with that of water extraction [39,40]. Microwave-assisted extraction is a novel, quick, and efficient method for extracting polysaccharides with higher bioactivities and a higher yield rate (41.6%) than that of hot water extraction (28.5%) [45]. For two other Panax species, water extraction has been used to isolate crude polysaccharides, and alkaline extraction [38,50,51], ethanol extraction [34,52], or ultrasonic extraction [43] have seldomly been used.

Current studies demonstrate that the polysaccharide extraction rates for American ginseng and notoginseng by alkali or methanol ranged from 1.8% to 2.8% [51]. Based on these findings, we conclude that different polysaccharides from three species can be isolated with different reagents, enzymes, or equipment to avoid the disadvantages of extraction processes and obtain high-yield and high-activity polysaccharides. Importantly, a combined method might be a more optimal strategy for extracting polysaccharides with different structures and bioactivities.

### 2.2. Separation and Purification

After ethanol precipitation and deproteinization, crude polysaccharides are purified and fractionated by column chromatography and membrane separation technology [53]. Column chromatography methods, including gel column chromatography and ion-exchange chromatography, are commonly used to purify the polysaccharides from three Panax species [39,47,54]. Gel column chromatography, which includes dextran- and agarose-gel columns, acts as a molecular sieve and separates polysaccharide molecules according to their size and shape [39,55]. As previously reported, the polysaccharides from ginseng were purified using Sephadex G-25 or G-75 columns [39,55]. A study showed that four polysaccharides from notoginseng were purified using gel filtration chromatography [56]. For ion-exchange chromatographic separation, an anion-exchange column packed with diethylaminoethyl (DEAE)-cellulose, DEAE-dextran gel, or DEAE-agarose gel has been the most used to separate ginseng polysaccharides. These materials possess different advantages, including large adsorption capacity, strong stability, fast elution, and weak protein binding [53].

To be specific, the crude polysaccharides from ginseng can be purified on a DEAE Sepharose Fast Flow column, Sephadex G-75 [39], Sephadex G-100 [52], Sepharose CL-6B [34], or Sephacryl S-200 column [34], which are eluted with distilled water and different concentrations of stepwise NaCl solution. The alkali-extractable polysaccharides from North American ginseng have been purified with a DEAE Sepharose Fast Flow column [38] or DEAE-Sepharose CL-6B column [57]. After extraction with 1 M KOH, the polysaccharide fraction for notoginseng was applied to a DEAE-Sepharose CL-6B column, followed by gel-permeation chromatography for purification [58]. Further purification to remove oligosaccharides can be accomplished using a dialysis membrane (3.5 × 10^3^ Da or 1 × 10^3^ Da) based on the concentration difference [30,39,59,60]. In addition, ultrafiltration mainly separates starch and protein from the polysaccharide fractions of the three species, and this is suitable for the large-scale purification of polysaccharides [61].

**Figure 1:**
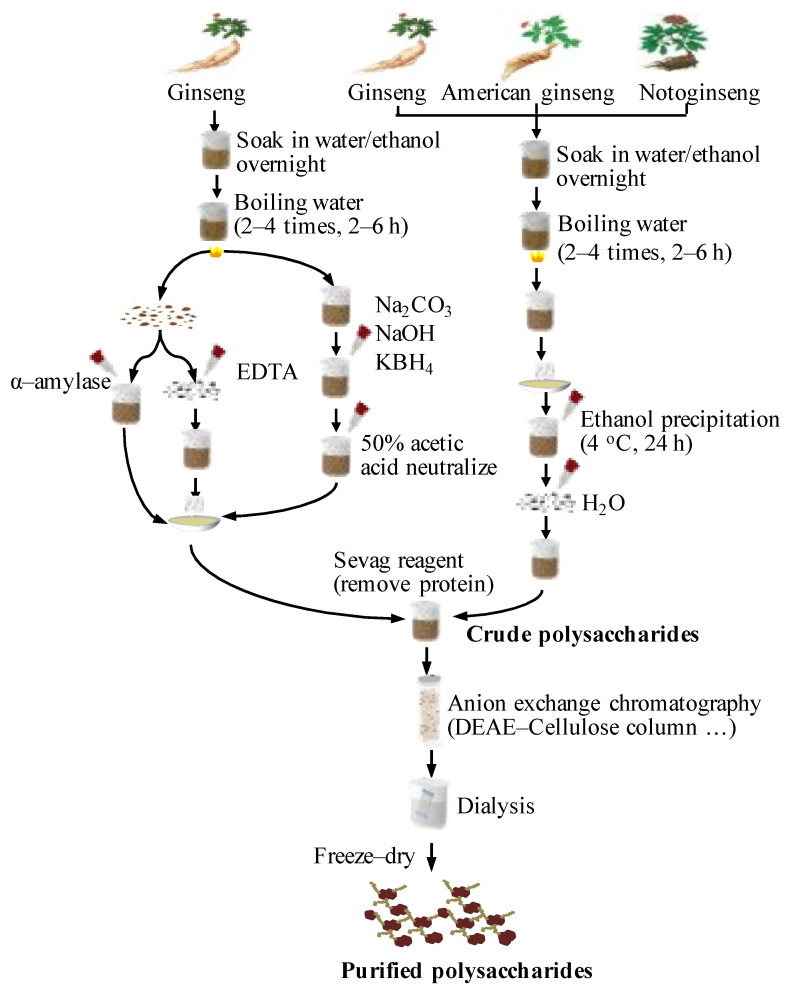
Schematic procedures for extraction and purification of the polysaccharides from three Panax species.

Collectively, combined purification methods have been used to obtain different fractions of the polysaccharides from three common Panax species, according to experimental aims. To analyze the structures and activities of polysaccharides of three species, polysaccharide fractions should be commonly extracted using hot water or alkali, then purification should proceed using column chromatography and membrane separation technology to obtain target polysaccharides. A procedural comparison of the extraction and purification techniques for different polysaccharides from three Panax species is shown in Figure 1.

## 3. Structural Characteristics

The polysaccharides from the three species are natural polymers of more than 10 monosaccharides consisting of linear or branched carbohydrate chains joined by glycosidic linkages [62]. Owing to the complexity and diversity, chemical methods (acid hydrolysis, periodate oxidation, and Smith degradation) and physical methods (nuclear magnetic resonance spectroscopy and mass spectrometry) are used to analyze polysaccharide composition characteristics and primary structures, including monosaccharide composition, the sequence and linkage of sugar groups, and anomeric carbon or sugar ring configuration [9,10,40]. Currently, enzyme-linked immunoassay, electron scanning microscopy, and circular dichroism have been used to determine advanced structures, which is a challenge in the research area of polysaccharide structure [57]. Neutral polysaccharides are the main components of ginseng polysaccharides and acid polysaccharides are a small portion and include pectin-containing rhamnose (Rha) and homogalacturonic acid [63]. Ginseng pectin, an acid polysaccharide mixture, is comprehensively studied to explore its composition and structure, which contains galactose (Gal), galacturonic acid (GalA), arabinose (Ara), and Rha [34,64]. Polysaccharide composition and structure are analyzed by a series of methods and techniques, but it is difficult to identify advanced structures such as hydrogen bonding of polysaccharide main chains, repeating sequences of sugar chains, and non-covalent bonding of polymer chains.

### 3.1. Polysaccharides from Ginseng

The composition and structural characteristics of ginseng polysaccharides have been extensively summarized in recent reviews [9,10]. Based on these reports by Zhao, and Guo, we summarized the updated publications describing the structural characteristics of ginseng polysaccharides and show new detailed results from recent reports, which are listed in Table 1. Kim et al. purified white ginseng neutral polysaccharide (WGNP) and white ginseng acidic polysaccharide (WGAP), which contain different amounts of carbohydrate and uronic acid [31]; there is a greater amount of uronic acid in WGNP (25.7%) than that in WGAP (0.8%). High-performance gel permeation chromatography (HPGPC) analysis showed that the molecular weights of two polysaccharide fractions, WGNP and WGAP, are estimated to be 16.1–70.4 kDa and 50.0–80.0 kDa, respectively [31]. It was also reported that other ginseng polysaccharides contained an α-(1→4)-GalA backbone (homogalacturonan, HG), rhamnogalacturonan I (RG-I)-rich pectin, arabinogalactans, and different molecular weights and sugar compositions [35,48,59,65,66]. As reported, a purified extract of ginseng berry polysaccharide (GBPP) with 12% yield is composed of Gal (26.6%), Glc (5.4%), Ara (19.5%), Rha (8.4%), Man (1.5%), GalA (15.2%), and Xyl (2.2%) and presents a moiety of arabino-β-3,6-galactan (35%) [18]. The chemical characteristics of three major fractions from GBPP demonstrate that GBPP-I (76 kDa) consists of 89.1% neutral sugars, GBPP-II (11 kDa) is composed of RG-II and other polysaccharides (61.3% neutral sugars and a Kdo-like material with unusual sugars) and GBPP-III (2.2 kDa) is enriched with phenolic compounds and sugars (hydrolyzed pectic polysaccharides) [19]. Another study of neutral polysaccharides from ginseng shows that GPNE-I (80.3 kDa) consists of a glucan domain and type I and II arabinogalactans (AG-I, AG-II), and GPNE-II (31.5 kDa) consists of a (1→4)-α-d-Glcp backbone and I and II exhibit different branching degrees at 38.17% and 50.78%, respectively [20]. The proposed structure of a low-molecular weight polysaccharide of ginseng, MCGP-L, was clearly elucidated by monosaccharide composition and methylation analyses and NMR spectroscopy [55]. Furthermore, different ranges of molecular weight, various monosaccharide compositions, HG backbone with hairy regions of RG-I, α-dominating configurations, and (1→)- or (1→6)- and (1→3)-glycosidic linkages were found in other ginseng polysaccharides extracted by hot water [27,34,36,52,54,67,68,69,70]. It was determined that RG-I, xylan, and xyloglucan are major components in ginseng polysaccharides sequentially extracted by Na_2_CO_3_ [37]. In addition, when polysaccharide fractions from ginseng were extracted by dialysis (12–14 kDa), different ratios of Ara, Fuc, Xyl, Rha, Man, Glc, and Gal were measured [41,71,72,73]. For ginseng polysaccharides obtained from ethanol and enzyme-assisted extraction and Sephadex G-100 column purification, RGP-AP-I is composed of 9.5% Rha, 18.4% GalA, 30.4% Gal, and 35.0% Ara and comprises the RG-I structure with a repeating linkage unit, →2)-Rhap-(1→4)-GalAp-(1→, (1→5)-linked arabinan, (1→4)-linked galactan, and arabino-β-3,6-galactan [22]. For ginseng polysaccharides extracted by enzymes (α-amylase, cellulase, EDTA, RNase, or DNase), Gal, Glc, Ara, Rha, Fuc, Man, GalA, and GlcA were identified and starch-like glucan, HG, and RG-I pectin were abundant in these fractions [40,42,74]. The polysaccharides from ginseng hydrolyzed by endo-1,4-β-d-galactanase mainly contain RG-I, RG-II, and AG-I domains and consist of Gal, Glc, Ara, Rha, Man, GalA, Fuc, and GlcA at different ratios [75,76].

### 3.2. Polysaccharides from American Ginseng

As published in 2020, the structural characteristics and immunomodulatory properties of American ginseng polysaccharides have been summarized [21]. In this review, we summarize the structural features of polysaccharides from American ginseng from all publications until April 2021, which are listed in Table 2. An extraction was performed for 25 similar polysaccharides from American ginseng using hot water or 0.3 M NaOH, and DEAE-Sepharose or Sephacryl S-300 columns were used for purification. The structural characteristics of five main fractions from American ginseng polysaccharides were elucidated by multiple techniques, which demonstrates that they have different molecular weights, ranging from 3.1 × 10^3^ to 9.7 × 10^6^ Da, as well as different sugar components, consisting of Ara, Rha, Xyl, Man, Gal, Glc, GalA, and GlcA [57]. NMR spectroscopy showed that these five polysaccharides mainly contain (1→6)-α-d-Glcp, (1→5)-α-l-Araf, (1→4)-β-d-Rhap, (1→4)-α-d-Manp, β-d-Galp, and β-d-xylose [57]. Non-starch polysaccharides from the roots of American ginseng (GSP) consist of Rha, Ara, Gal, Glu, and uronic acid with a weight ratio of 1:4:8:8:50 and contain a major sugar residue (4-α-d-Gal*p*A) and other residues (2-α-l-Rha*p*, 2,4-α-l-Rha*p*, α-l-Ara*f*, β-d-Gal*p*, and 4-β-d-Gal*p*), which suggests that GSP is a pectin molecule with HG and a small portion of RG-I [77]. AGC1 is a neutral polysaccharide isolated from suspension cultures of American ginseng. AGC1 has an average molecular weight of 5.2 kDa and is composed of >60% Gal and other neutral sugars (Ara, Xyl, Glu, Man, and Rha) and primarily contains 48.5% 3-linked Gal*p*, 10.2% 3,6-linked Gal*p*, 5.2% terminal, and 4.4% 6-linked galactopyranosyl residues [78]. Moreover, the major glycosidic linkages, including terminal (47.7%), 4-linked (15.6%), and 6-linked (8.1%) galactopyranosyl residues, 2,4-linked rhamnopyranosyl residue (8.1%), and 4-linked galacturonopyranosyl residue (6.8%), were found in an acidic polysaccharide, AGC3, with the presence of RG-I pectin [79]. Moreover, other polysaccharides from hot water extraction have also been reported to examine their molecular weight, sugar composition, and structural features [51,80,81,82,83]. After extraction of American ginseng root with 0.3 M NaOH, fractions AEP-1 and AEP-2 were obtained, which consist of Gal, Glc, and GalA with a molar ratio of 0.97:4.67:3.92 and Gal, Glc, Ara, Man, and GalA with a molar ratio of 1.68:3.02:1.03:0.76:3.65, respectively [38]. In addition, microwave-assisted extraction of American ginseng root yielded specific polysaccharides from different locations that contain Rha, Ara, GalA, Man, Glc, and Gal with different ratios of monosaccharide composition [61]. A new report demonstrated that polysaccharides from American ginseng extracted by ultrasonication consist of Ara, Rha, GalA, Man, Glc, and Gal with a ratio of 31:4:1:2:72:59 and present 1→4 glycosidic linkages and a →4)-GalA-(1→ moiety [30]. Based on current findings, there are fewer articles on American ginseng polysaccharides than those on ginseng polysaccharides. Importantly, American ginseng polysaccharides should be further investigated in the future to elucidate their chemical structures as analytical techniques progressively improve.

### 3.3. Polysaccharides from Notoginseng

The structural characteristics of notoginseng polysaccharides have been studied for more than 30 years. In 1987, a polysaccharide with a molecular mass of 1.50 × 10^6^ Da, sanchinan-A, was examined to determine whether terminal arabinofuranosyl, terminal galactopyranosyl, and 3-, 6-, and 3,6-linked galactopyranosyl residues were present [84]. In 1996, it was determined that four polysaccharides were heteroglycans with molecular weights of 37–760 kDa and were composed of Glu, Gal, Ara, Man, and Xyl in different molar ratios [56]. A fraction from the root of notoginseng called AIR contains cellulose and pectic polysaccharides, including 24% HG and 2% acidic RG-I, with high levels of type I 4-galactans and low levels of xyloglucan [50]. Fr, a fraction from AIR extracted with 1 M KOH, is composed of 4-galactans, heteroxylans, and small amounts of arabinan and HG. Its acidic fraction, 1MD3-G2, has a molecular weight of 1.14 × 10^6^ Da and contains 45% type I 4-galactan, 10% HG, 11% arabinan, 28% heteroxylan, and 4% RG-I [58]. It was reported that an arabinoglucogalactan (1) from notoginseng roots possesses (1→3)-linked β-d-galactofuranosyl and α-l-Ara f-(1→4)-β-d-Glcp-(1→ residues [85]. Three polysaccharides from different parts of *P. notoginseng*, MRP, BRP, and FRP, have different chemical and monosaccharide compositions [26]. RN1 is an arabinogalactan polysaccharide and possesses a backbone of 1,6-linked Galp and branches of 1,5-linked, 1,3,5-linked, terminal Ara, and terminal Gal [86]. Notoginseng polysaccharides from different locations were obtained by microwave-assisted extraction. They were named N1–N5, and contain Rha, Ara, GalA, Man, Glc, and Gal, which contain 11.9–15.0 mg/g of specific polysaccharides, as determined by the HPSEC-MALLS-RID method [61]. Two novel polysaccharides, MAP5A (113.8 kDa) and MRP5 (91.6 kDa), are composed of Gal, Glu, Ara, and Rha in different ratios. MRP5 contains →3)-β-Glcp-(1→, →3)-β-Galp-(1→, →3,6)-β-Galp-(1→, →3)-β-Galp-(1→, →3,6)-β-Galp-(1→, →3)-α-Rhap-(1→, →3)-α-Araf-(1→, and α-Araf-(1→ repeating residues [87]. PNPS-0.5M is a novel acidic polysaccharide that is a notoginseng residue and it contains a backbone of (4→1)-linked GalA, (1→)-, (5→1)-linked Araƒ, and (1→)-linked Rhap branches [43]. PNPS-0.3 is another polysaccharide with a molecular weight of 76.655 kDa that mainly consists of a backbone of →4)-α-d-GalAp-(1→4-β-l-Rhap-1→4)-β-d-Galp-(1→ residues and a branch of α-l-Araf-1→5)-α-l-Araf-(1→ [32]. A new study to isolate water-soluble polysaccharides from notoginseng discovered a starch-like polysaccharide, PNPN, and its pectin fractions. Its structural characteristics were analyzed using multiple methods and it contains Gal, Ara, GalA, and Rha, with different sugar compositions and molecular weights [29]. Among these sub-fractions, two fractions mainly include 1,4-β-d-galactans, 1,5-α-l-arabinan, and AG-II and three fractions consist of HG, together with different ratios of RG-I and RG-II domains [28]. Structural fingerprinting analysis suggests that PPN, a crude polysaccharide from notoginseng extracted by ultrasonication, contains Ara, GalA, Man, Glc, and Gal, with a molar ratio of 2:1:2:83:7, contains 1→4 glycosidic linkages and shows an abundant amount of →4)-Hexp-(1→ [30]. For neutral polysaccharides from notoginseng, the molecular weights of five polysaccharides ranged from 1.81 × 10^4^ to 10.56 × 10^4^ Da [2]. In addition, a fermented polysaccharide from notoginseng exhibited higher levels of polysaccharides, compared with that extracted by water, but further investigation is required to determine its structural characteristics [2]. Collectively, 36 polysaccharides from notoginseng have already been found and their molecular weights, composition, and structural features have been summarized in Table 3. A future research direction should be to obtain the detailed structural characteristics, such as the backbone, different branches, and glycosidic linkages, of different notoginseng polysaccharides.

To further compare the similarities and differences of the polysaccharides from three Panax species, we summarized the extraction method, purification method, molecular weight, monosaccharide composition, and structural characteristics of these polysaccharides in Table 4. Ginseng polysaccharides have been extensively studied for many years, and the polysaccharides from the other two species have been recently investigated. Hot water, alkali, enzymes, EDTA, and microwaves are commonly used methods for extracting polysaccharides, especially from ginseng samples. Recently, ultrasonication was used to extract and obtain polysaccharide fragments from three Panax species. Moreover, DEAE-cellulose, Sepharose G-50 and CL-6B columns, dialysis, and ultrafiltration are used for purification to isolate different fragments of the polysaccharides from three Panax species. Importantly, the polysaccharides from American ginseng and notoginseng should be highly purified using different purification methods. The molecular weights of these polysaccharides encompass very broad ranges with different sizes, which might be related to the methods used for extraction and purification. For the monosaccharide composition, Ara, Gal, GalA, Glc, GlcA, Man, and Rha are commonly found in the polysaccharides from the three Panax species; Arab, Fru, Fuc, Rib, and Xyl were only discovered in ginseng polysaccharides. AG-I, AG-II, HG, RG-I, RG-II, and glycosidic linkages are extensively found in ginseng polysaccharides. The polysaccharides from the other two species consist of RG-II, galactan, and arabinopyranosyl residues, which should be further investigated. Collectively, the structural features of the polysaccharides from the three species, especially American ginseng and notoginseng polysaccharides, should be explored using a series of new techniques.

Briefly, the differences and similarities among these three common Panax species polysaccharides are listed as follows. Similarities: Most of the three common Panax species polysaccharides are extracted by hot water extraction. Some monosaccharides exist in all three Panax species polysaccharides, such as Ara, Gal, GalA, Glc, GlcA, Man, and Rha. RG-II and 1→4 glycosidic linkages as linear backbone are extensively found in three common Panax species. In addition, the activity research of three common Panax species polysaccharides focuses on antitumor activity, immunomodulatory activity anti-oxidative activity. Differences: Nowadays, the ginseng polysaccharides that have been reported could be extracted by enzyme, EDTA, microwave, ultrasonication, and other new methods. While American ginseng and notoginseng polysaccharides only recently began to use new extraction methods. The molecular weights of three common Panax ginseng species polysaccharides are different, which may be related to the extraction and purification methods. The structure of ginseng polysaccharide is complex and diverse, while American ginseng and Panax notoginseng are rather simple. In addition to anti-tumor activity, immunomodulatory, and anti-oxidative activity, ginseng polysaccharide also has antihyperglycemic activity and anti-fatigue activity, and prolongs life. Panax notoginseng can protect the liver. The activity of American ginseng is mainly immunomodulatory and other active polysaccharides are less studied. Otherwise, there is no relevant study comparing the efficacy of these three common Panax species polysaccharides, at present.

## 4. Biological Activities of Different Polysaccharides from the Three Species

### 4.1. Antitumor Activity

Many studies have shown that polysaccharides from the *Araliaceae* family, especially ginseng polysaccharides, exhibit anti-tumor activity in cell and animal models. In peritoneal macrophage and leukemia cell models, ginseng polysaccharide (GPS) stimulated macrophages to increase the levels of cytokines, including tumor necrosis factor-α (TNF-α), interleukin-1 (IL-1), IL-6, and nitric oxide (NO) production against leukemia [88]. In malondialdehyde (MDA)-MB-231 breast cancer cells, GPS activated p65-IKZF1 signaling and apoptosis to inhibit cell proliferation [89]. In HCT-116 and HT-29 human colon cancer cells, ginseng berry polysaccharide extract (GBPE) and its purified fragment, GBPP, significantly inhibited IL-8 secretion and Th1 and Treg cell differentiation to suppress cell growth [90]. In B16-BL6 melanoma, GBPP exhibited a function similar to that observed in colon cancer [18]. Similarly, NFP, which is a polysaccharide from Korean red ginseng, inhibited melanoma cell metastasis to the lung, which may have resulted due to its immunity-enhancing effect [91]. Furthermore, a polysaccharide from ginseng leaves, GS-P, promoted macrophages and natural killer (NK) cells to exhibit anti-metastatic activity against colon cancer [92].

A neutral polysaccharide, WGPN, exhibited functions similar to those of GPS and, when combined with 5-fluorouracil, a synergistic effect was observed, which indicates that it has potential as an adjuvant that can be used against sarcoma tumors [93]. An acidic polysaccharide, WGPA, and its fraction, WGPA-3-HG, inhibited HT-29 colon cancer cell proliferation and caused G_2_/M phase arrest [94]. Moreover, temperature-modified WGPA-3-HG (MWGPA-3-HG) increased the percentages of S and G_2_/M phase cells and induced apoptosis by activating caspase-3 [94]. In gastric cancer cells, PGPW1 regulated Twist expression to block metastasis [36]. In a mouse model of Lewis lung carcinoma, two ginseng polysaccharides (GP and GFP1) significantly increased the ratio of CD4^+^/CD8^+^ T lymphocytes and promoted NK cytolytic activity, which shows that they possess a satisfactory immunomodulatory effect against lung cancer [95,96]. In addition, serum analysis demonstrated that GPS increased Th1 cytokines (INF-γ and IL-2) and decreased Th2 cytokines (IL-4 and IL-5) in 96 patients with non-small-cell lung cancer [97]. A new report demonstrated that ginseng polysaccharides altered the gut microbiota and the kynurenine/tryptophan ratio to potentiate the anti-tumor effect of anti-PD-1/PD-L1 immunotherapy [98].

For American ginseng polysaccharides, only one study showed that ginseng polysaccharide nanoparticles (GPS-NPs) inhibited oxidative damage and skin cancer induced by UVB exposure [82]. Three studies were reported that tested notoginseng polysaccharides against pancreatic and hepatic cancers. In a BxPC-3 pancreatic cancer xenograft model, an arabinogalactan polysaccharide (RN1) from notoginseng flowers inhibited microvessel formation and migration by the inhibition of BMP2/Smad1/5/8/Id1 signaling [86]. In H22 cells and a tumor-bearing mouse model, PPN, a notoginseng polysaccharide, activated CD4^+^ T-cells and elevated serum IL-2 to inhibit liver cancer growth and prolong survival [99]. Furthermore, a neutral notoginseng polysaccharide, NPPN, was combined with CTX and significantly inhibited H22 tumor growth via myelosuppression [100]. The mode of action of different ginseng polysaccharides is mainly that they regulate immune cytokines to inhibit tumor progression. Because there are few studies that have reported that the polysaccharides from the other two species exhibit anti-cancer activities, this could be a potential research direction.

### 4.2. Immunomodulatory Activity

Many studies have shown that different ginseng polysaccharides possess immunomodulatory functions against numerous immune-related diseases. Two ginseng polysaccharides named FGWP-CA and GPS increased the NK cell activity induced by CTX and played a role in immune regulation [40,101]. In an autoimmune encephalomyelitis mouse model, APG, which is an acidic polysaccharide from ginseng, promoted Treg cell generation through Foxp3 activation and the production of inflammatory cytokines, such as interferon-γ (IFN-γ), IL-1β, and IL-17 [102]. This might also modulate the infiltration of CD4^+^ T cells and CD11b^+^ macrophages into the spinal cord [103]. In different cell models, four ginseng polysaccharides (GPNE-I, WGPA-2-RG, ginsan, and PS-NPs) enhanced lymphocyte proliferation, macrophage phagocytosis, and dendritic cell maturation by regulating various cytokine levels and NO production [20,23,35,64,104]. Several studies reported that RGAP, an acidic polysaccharide from Korean red ginseng, activated the extracellular signal-regulated kinase (ERK)/c-Jun N-terminal kinase (JNK) and nuclear factor kappa B (NF-κB)/AP-1 signaling pathways and augmented the production of IL-6, IL-12, TNF-α, and NO in macrophages from a female BALB/c mouse model [49,105]. Moreover, RGAP increased the numbers of T cells, B cells, macrophages, and IgM antibody-forming cells to enhance macrophage phagocytosis activity [106] and increase the number of plaque-forming cells [107]. RGP-AP-I and RG-CW-EZ-CP are polysaccharides that exhibit functions and molecular mechanisms that are similar to those of RGAP [22,41]. Furthermore, a neutral ginseng polysaccharide (NGP) stimulated the maturation of bone marrow dendritic cells through upregulation of MHC class II, CD80, and CD86 [108]. In addition, PGP-SL and GMP are two polysaccharides that exhibited immunopotentiation effects by enhancing the Ca^2+^/calcineurin/NFAT signaling pathway in spleen lymphocytes [109] and reactive oxygen intermediates in peritoneal macrophages [110], respectively. In weaned piglets induced with lipopolysaccharide (LPS), GPS regulated the TLR4/MyD88-NF-κB pathway to reduce immunological stress [111].

For the polysaccharides from American ginseng, high-molecular weight polysaccharides can trigger the ERK, PI3K, p38, and NF-κB signaling pathways to immunomodulate human peripheral blood mononuclear cells [112]. In LPS-induced rats and alveolar macrophage models, American ginseng root polysaccharides (AGRPSs) exerted an immunomodulating effect under normal conditions and suppressed the immune response induced by LPS by regulating NO and TNF-α levels [113]. Experiments were conducted with five fractions of American ginseng polysaccharides (WPS-1, WPS-2, SPS-1, SPS-2, and SPS-3) to demonstrate increases in macrophage phagocytosis, NO production, and splenic lymphocyte proliferation [57]. In a CTX-induced immunosuppressive mouse model, American ginseng polysaccharide (AGP) enhanced CD4^+^ T cells and IgA-secreting cells and regulated the gut microbiota to prevent side effects from cancer chemotherapy [114]. AGPs extracted by ultrasonication exhibited satisfactory immunostimulant activity by upregulating NO and cytokine production [115]. Two acidic polysaccharide fractions, named AGC1 and AGC3, enhanced IL-6, TNF-α, granulocyte-macrophage colony stimulating factor (GM-CSF), and monocyte chemoattractant protein-1 (MCP-1) levels by regulating the NF-κB (p65/RelA) and p38 signaling pathways in RAW 264.7 macrophages and primary murine splenocyte models [78,79]. In addition, five polysaccharides (PPQA2, PPQA4, PPQA5, PPQN, and GPS NPs) regulated the production of cytokines, including TNF-α, IL-1β, and IL-6, to demonstrate their immunomodulating abilities in the presence of RAW264.7 macrophages [51,80,116]. PBGA12, which is a notoginseng polysaccharide, enhanced IFN-γ and TNF-α so that they stimulated the complement system [56]. Another polysaccharide fraction from notoginseng, Fr1MKOH, exhibited complement-fixing activity and a mitogenic effect on human polymorphonuclear neutrophils [58]. The novel polysaccharide PNPS-0.3 upregulated the amounts of cytokines, such as TNF-α, IL-12, and induced a T-cell immune response (CD4, CD8, CD69, and MHC II) with increased INF-β secretion by triggering the TLR4/TLR2-NF-κB signaling pathway in bone marrow dendritic cells [32]. The polysaccharides from the three Panax species mainly enhance immune cell proliferation (lymphocytes, macrophages, NK cells, and dendritic cells) and promote the production of multiple cytokines (IL-6, IL-12, IFN-γ, and TNF-α) by regulating the ERK/JNK/p38, TLR4/TLR2-NF-κB, and Ca^2+^/calcineurin/NFAT pathways.

### 4.3. Anti-Oxidative Activity

At present, there is a greater number of reports on the anti-oxidant activity of ginseng polysaccharides than that of reports on American ginseng and notoginseng polysaccharides. Three ginseng polysaccharides, named ginseng-SDF, native ginseng polysaccharide, and ginseng polysaccharide, significantly scavenged 1,1-diphenyl-2-picrylhydrazyl (DPPH), hydroxyl, or superoxide anion radicals to exhibit their anti-oxidant activities [24,25,117]. Neutral and acidic polysaccharides from ginseng (WGNP and WGAP) are antioxidants that scavenged hydroxyl radicals and decreased reactive oxygen species (ROS) and lipid peroxidation in a *Caenorhabditi*s *elegans* model [31]. In a streptozotocin-induced diabetic mouse model, neutral and acidic ginseng polysaccharides (WGPN and WGPA) exhibited anti-diabetic potential, which was mediated by the inhibition of anti-oxidative activity [35]. WGPA and its fraction, WGPA-A, inhibited oxidative stress by regulating the balance of oxidation and anti-oxidation, which resulted in an anti-fatigue effect [59,118]. AEP-2 is an alkali-extractable polysaccharide from American ginseng that exhibited higher values of Trolox equivalent and oxygen radical antioxidant capacities [38]. A polysaccharide from notoginseng (FPNP) decreased the amount of MDA and increased the activities of antioxidant enzymes, such as catalase (CAT), glutathione peroxidase (GSH-Px), and superoxide dismutase (SOD), by activating the transforming growth factor-β (TGF-β)/Smad signaling pathway into H_2_O_2_-induced human dermal fibroblast cells [2]. Collectively, nine polysaccharides from three Panax species have been reported as natural antioxidants.

### 4.4. Other Biological Functions

In addition to the main functions above, other biological functions of different polysaccharides from ginseng and notoginseng have been reported in recent years. GPS promoted food intake in mice, which may be related to appetite-regulation peptides and circulating glucose levels [119]. In an ethanol-induced gastrically injured rat model, GPS increased anti-oxidant activity and suppressed inflammation [27]. In a diarrhea mouse model induced by antibiotics, a ginseng polysaccharide named WGP changed the gut microbiota composition and diversity and balanced metabolic processes to recover the mucosal structure [69]. Moreover, WGP prevented cisplatin-induced endoplasmic reticulum stress and cell death in renal cells by activating PERK-eIF2α-ATF4 signaling [120].

In a diabetic rat model, ginseng polysaccharide (GP) enhanced ginsenoside Rb1 biotransformation and this resulted in an anti-diabetic effect and protected against dextran sulphate sodium-induced colitis, which may be associated with the gut microbiota [121,122]. Similarly, ginseng polysaccharide APG protected the mouse small intestine from irradiation by inhibiting the p53-dependent and mitochondrial apoptosis pathways [123]. Furthermore, GP decreased lung viral titers and inhibited IL-6 to protect mice from H1NI influenza virus infection [124]. Additionally, an acidic polysaccharide fraction of ginseng (AP1) activated the reperfusion injury salvage kinase and endothelial nitric oxide synthase (eNOS)-dependent pathways to maintain mitochondrial function against myocardial hypoxia/reoxygenation injury [125].

**Figure 2:**
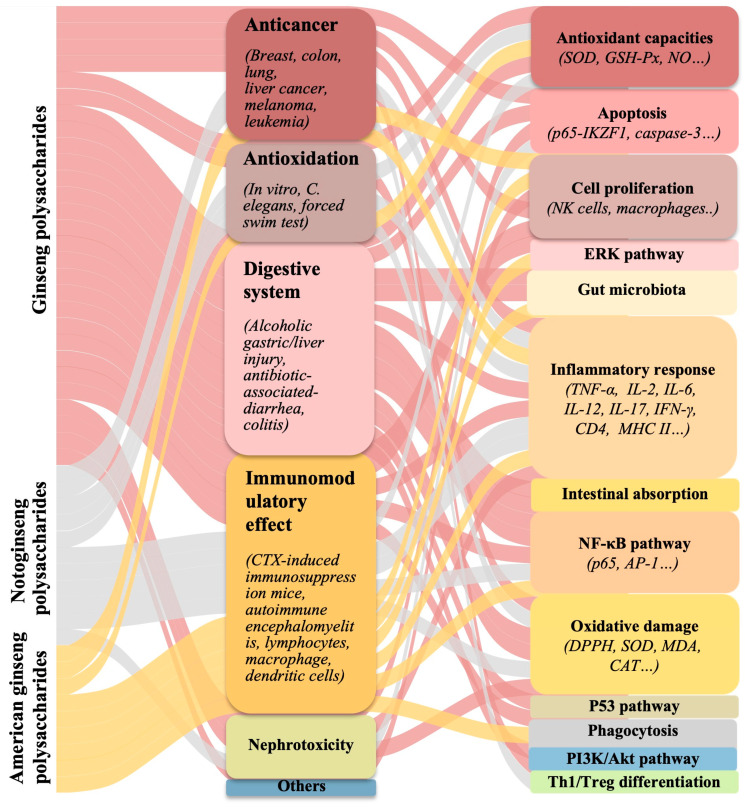
Summary for biological activities and molecular mechanisms of the polysaccharides from three species against multiple diseases.

For anxiety disorders, another acidic polysaccharide, WGPA, exhibited an anti-depressant-like effect by affecting social interactions and aggressive behaviors in mice [65]. For skin health, a byproduct polysaccharide from red ginseng was processed by enzyme-linked high pressure to produce ELHPP-RGBPs, which inhibited the AP-1/MMP-1 pathway and prevented solar ultraviolet-induced skin wrinkles and atopic dermatitis [126]. The notoginseng polysaccharides PNPS and PNPS-0.5M inhibited a caspase-3 cascade or regulated the alcohol dehydrogenase pathway to protect from cerebral ischemia/reperfusion injury or alcoholic liver damage, respectively [43,127]. These three species of polysaccharides exhibited anti-tumor, immunoregulatory, and anti-oxidation activities, as well as anti-diabetic, anti-fatigue, and anti-depression activity. The biological functions, detailed models, and molecular mechanisms of the polysaccharides from the three Panax species are listed in Table 5 and are shown in Figure 2.

## 5. Conclusions and Future Perspective

**Figure 3:**
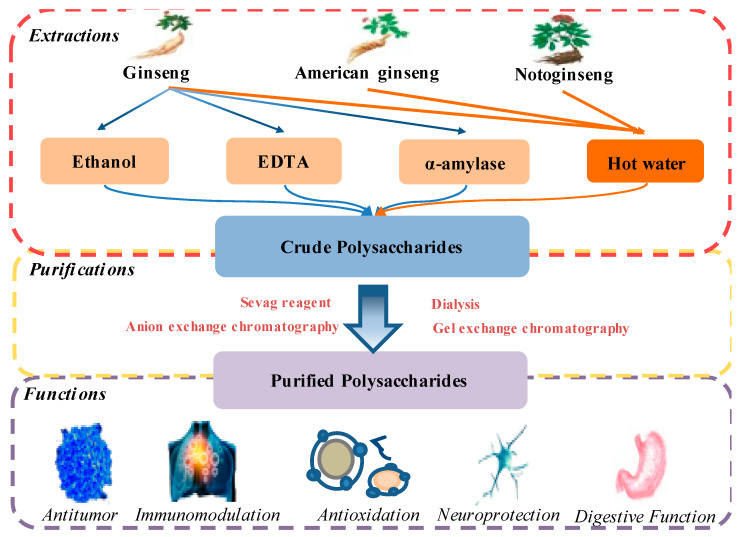
Summary for extraction, purification, and functions of the polysaccharides from three species.

This review summarized recent advances associated with 112 polysaccharides from ginseng, 25 polysaccharides from American ginseng, and 36 polysaccharides from notoginseng and compared the differences in extraction, purification, and structural features. Most studies focused on ginseng polysaccharides and, if comparisons were made, the polysaccharides used were from American ginseng and notoginseng. For the extraction, purification, and structural analysis of polysaccharides, the processes were similar for all three Panax species. Generally, ginseng (4–5-year-old, crushed and passed through 60 or 80 mesh sieves, dried at 60 °C and stored in the freezer), American ginseng (4–5-year-old, ground and passed through a 40-mesh, 60-mesh or 80-mesh sieve and dried at room temperature), notoginseng (dried at 60 °C for 24 h, ground and passed through a 60-mesh sieve and stored in a desiccator at room temperature). The greatest number of articles has been written on ginseng polysaccharides, followed by American ginseng and Panax notoginseng. They possess anti-tumor activity, immunoregulatory effects, anti-oxidant activity, and other pharmacological functions, which are mediated by multiple signaling pathways, including the MAPK, NF-κB, or redox balance pathways (Figure 3).

Seven important aspects should be further considered based on the recent findings for these polysaccharides from the three Panax species. (1) The structural characteristics and biological activities of the polysaccharides from American ginseng and notoginseng should be deeply investigated. (2) As shown in a recent report [128], new approaches, such as two-dimensional attenuated total reflection Fourier transform infrared spectroscopy based on a gradient heating program, should be developed to discriminate and identify the structural features of Panax polysaccharides. (3) The differences in biological activity might be related to functional groups, branching, and conformational characteristics of the polysaccharides, which could be a future direction for exploring the relationship between structure and activity. (4) The polysaccharides can accelerate the microbial metabolism of ginsenoside Rb1, suggesting the potential roles of the polysaccharides on the gut microbiota [114,121,122]. Studies on the effects of the polysaccharides on ginsenoside absorption in vivo should be strengthened in future. (5) The polysaccharides from American ginseng suspension culture [78,79] and ginseng polysaccharide nanoparticles have been heavily researched in recent studies [82,104,116] and these can provide new opportunities for further development of the polysaccharides with obvious pharmacological properties. (6) Currently, only ginseng polysaccharides have been evaluated to explore their efficacy and safety in healthy volunteers and patients with non-small cell lung cancer [97,129]. Clinical trials using the polysaccharides from three Panax species should be performed to determine their efficacies. (7) At present, we found that the articles on ginseng polysaccharides include experiments with ginseng or American ginseng, which are mostly grown in Jilin Province, China (34.8% and 50%, respectively). Most of the studies on Panax notoginseng originated from Yunnan Province, China (52%). Therefore, it was rare that articles mentioned information describing the cultivation period, storage conditions, or dryness; thus, we were not able to determine the relevant links between different conditions for different ginseng species. We suggest that a greater number of studies should focus on these Panax spp., because it could be meaningful to use Panax herbs of different ages and compare their effects on the isolation, purification, and activity of polysaccharides. Collectively, this review can provide new insights into the similarities and differences of the polysaccharides from three Panax species, which can facilitate and guide further studies to explore the properties of the *Araliaceae* family used in traditional Chinese medicine.

## Figures and Tables

**Table 1:** Structure and composition of ginseng polysaccharides.

No.	Polysaccharide Name	Extraction and Purification	Composition Ratio of Sugars (Mass Percentage or Molar Ratio)	Molecular Weight (Da)	Structure Feature	Refs
(1)	WGNP	Hot water, purification by DEAE-Cellulose, dialysis	Gal:Glu:Ara = 1.1:97.9:1.0	1.61–7.04 × 10^4^		[31]
(2)	WGAP	Hot water, purification by DEAE-Cellulose, dialysis	Gal:Glu:Ara:GalA:GluA = 24.4:24.0:18.1:32.2:1.3	5–8 × 10^4^		[31]
(3)	WGPN	Hot water, purification by DEAE-Cellulose	Gal:Glc:Ara = 3.3:95.3:1.3		Starch-like glucans	[35]
(4)	WGPA	Hot water, purification by DEAE-Cellulose	Gal:Glc:Ara:GalA = 17.1:18.0:15.5:44.2		HG and RG-I-rich pectin and arabinogalactan	[35]
(5)	WGPA	Hot water, purification by DEAE-Cellulose	Gal:Glc:Ara:Rha:GalA:GlcA= 18.0:18.5:15.5:2.5:44.2:1.3		RG-I, HG-rich pectin, and arabinogalactan	[65]
(6)	WGPA-A	Hot water, purification by DEAE-Cellulose twice	Gal:Glc:Ara:Rha:Man:GalA:GlcA = 13.3:3.7:22.7:6.0:0.5:51.7:2.2			[59]
(7)	WGPA-N	Hot water, purification by DEAE-Cellulose twice	Gal:Glc:Ara = 18:66.3:15.7		Starch-like glucans and AG	[59]
(8)	WGPA-1-RG	Hot water, purification by Sepharose CL-6B column, DEAE-Cellulose	Gal:Glc:Ara:Rha:Gla A = 56.2:3.5:34.0:0.2:1.8		AG with trace RG-I	[66]
(9)	WGPA-2-RG	Hot water, purification by Sepharose CL-6B column, DEAE-Cellulose	Gal:Glc:Ara:Rha:Gla A = 44.4:2.9:40.9:4.1:5.3		AG with minor RG-I	[66]
(10)	WGPA-3-RG	Hot water, purification by Sepharose CL-6B column, DEAE-Cellulose	Gal:Glc:Ara:Rha:Gla A = 29.0:3.2:38.0:7.3:20.2		HG- and RG-I-domains	[66]
(11)	WGPA-4-RG	Hot water, purification by Sepharose CL-6B column, DEAE-Cellulose	Gal:Glc:Ara:Rha:Gla A = 13.5:4.4:26.1:11.4:38.4		HG- and RG-I-domains	[66]
(12)	WGPA-1-HG	Hot water, purification by Sepharose CL-6B column, DEAE-Cellulose	Gal:Glc:Ara:Rha:Gla A = 15.2:7.6:7.1:3.6:62.4	3.5 × 10^3^	HG with minor RG-I	[66]
(13)	WGPA-2-HG	Hot water, purification by Sepharose CL-6B column, DEAE-Cellulose	Gal:Glc:Ara:Rha:Gla A = 5.1:1.9:4.6:3.0:83.6	6.5 × 10^3^	HG with minor RG-I	[66]
(14)	WGPA-3-HG	Hot water, purification by Sepharose CL-6B column, DEAE-Cellulose	Gal:Glc:Ara:Rha:Gla A = 3.5:1.3:2.2:1.5:90.9	1.6 × 10^4^	HG with trace RG-I	[66]
(15)	WGPA-4-HG	Hot water, purification by Sepharose CL-6B column, DEAE-Cellulose	Gal:Glc:Gla A = 5.9:2.0:92.1	4.5 × 10^4^	HG with little RG-I	[66]
(16)	WGPA-1-RG	Hot water, purification by DEAE-Cellulose, dialysis, Sepharose CL-6B column	Gal:Glc:Ara:Rha:Man:GalA:GlcA = 56.2:3.5:34.0:0.2:2.5:1.8:1.9	1.0 × 10^5^	Arabinogalactans containing RG-I domains	[48]
(17)	WGPA-2-RG	Hot water, purification by DEAE-Cellulose, dialysis, Sepharose CL-6B column	Gal:Glc:Ara:Rha:GalA:GlcA = 23.3:3.4:25.2:2.3:41.3:4.5	1.1 × 10^5^	Arabinogalactans containing RG-I domains	[48]
(18)	WGPA-1-HG	Hot water, purification by DEAE-Cellulose, dialysis, Sepharose CL-6B column	Gal:Glc:Ara:Rha:Man:GalA:GlcA = 15.2:7.6:7.1:1.6:3.6:62.4:4.5	3.5 × 10^3^	α-(1→4)-GalA backbone (HG)	[48]
(19)	WGPA-2-HG	Hot water, purification by DEAE-Cellulose, dialysis, Sepharose CL-6B column	Gal:Glc:Ara:Rha:Man:GalA:GlcA = 5.1:1.9:4.6:3.0:0.2:83.6:1.6	6.5 × 10^3^	α-(1→4)-GalA backbone (HG)	[48]
(20)	WGPA-3-HG	Hot water, purification by DEAE-Cellulose, dialysis, Sepharose CL-6B column	Gal:Glc:Ara:Rha:Man:GalA:GlcA = 3.5:1.3:2.2:1.5:90.9:0.5	1.6 × 10^4^	α-(1→4)-GalA backbone (HG)	[48]
(21)	WGPA-4-HG	Hot water, purification by DEAE-Cellulose, dialysis, Sepharose CL-6B column	Gal:Glc:GlcA = 5.2:2.0:92.1	4.5 × 10^4^	α-(1→4)-GalA backbone (HG)	[48]
(22)	GBPP	Hot water, purification by dialysis (20 kDa)	Gal:Glc:Ara:Rha:Man:GalA:Xyl = 26.6:5.4:19.5:8.4:1.5:15.2:2.2		AG-II	[18]
(23)	GBPP-I	Hot water, purification by dialysis (20 kDa), Sephadex G-75, dialysis (12–14 kDa)	Gal:Ara:GalA:Xyl:Glu = 46.9:27.5:10.4:0.4:5.4	7.6 × 10^4^		[19]
(24)	GBPP-II	Hot water, purification by dialysis (20 kDa), Sephadex G-75, dialysis (12–14 kDa)	Gal:Ara:GalA:Rha:Glu:Xyl = 26.2:14.2:16.1:11.3:4.8:1.8	1.1 × 10^4^	RG-II and other polysaccharides	[19]
(25)	GBPP-III	Hot water, purification by dialysis (20 kDa), Sephadex G-75, dialysis (12–14 kDa)	Gal:Ara:GalA:Rha:Glu:Xyl = 23.4:6.9:13.0:5.5:8.1:7.8	2.2 × 10^3^	Phenolic compounds and sugars, eight different sugars of different composition	[19]
(26)	GPNE-I	Hot water, purification on a Sephadex G-100 column, DEAE-Cellulose	Gal:Glc:Ara = 3.7:37.8:1	8.03 × 10^4^	AG-I and AG-II	[20]
(27)	GPNE-II	Hot water, purification on a Sephadex G-100 column, DEAE-Cellulose	Gal:Glc:Ara = 1.9:7:1	3.15 × 10^4^	(1→4)-α-d- Glcp backbone with a substitution at O-6 on every two residues. (1→3)-α-d-Glcp and (1→6)-α-d-Glcp	[20]
(28)	WGPA-UN-N1	Hot water, purification on DEAE-Cellulose, Sephadex G-25 column, Sephadex G-50 column	Glc = 97.5	1.7 × 10^4^	α-(1→6)-d-glucan	[73]
(29)	WGP	Hot water	Glu:Gal:Ara:GalA:Rha:Man = 76.7:6.5:5.1:9.2:1.4:1.1		Neutral glucan-like polysaccharides, and contains some amount of RG-I and HG	[69]
(30)	MCGP-L	Hot water, purification by Sephadex G-25 column	Gal:Glc:Man = 1:14.8:1.2	3 × 10^3^	(1→4)-linked-α-d-Glcp residues and with branch chain substituted at O-6 position of (1→4,6)-linked-α-d-Glcp. The branch chain consists of →6)-α-d- Galp-(1→, →2)-α-d-Manp-(1→ and β-d-Glcp-(1→	[55]
(31)	WPPG	Hot water, purification by DEAE-Cellulose column (Microwave-assisted)	Gal:Glu:Rha:Man:GalA:Rib:Arab = 1.56:67.6:1.09:3.75:1:3.42:1	2.07 × 10^6^		[45]
(32)	BGP-60	Hot water, purification on Sephadex G-75 and DEAE-Cellulose columns	Gal:Glc = 22.3:77	2.86 × 10^4^	α-Dominating configurations	[67]
(33)	BGP-65	Hot water, purification on Sephadex G-75 and DEAE-Cellulose columns	Gal:Glc:Ara = 37.05:59.23:3.72	2.67 × 10^4^	α-Dominating configurations	[67]
(34)	BGP-70	Hot water, purification on Sephadex G-75 and DEAE-Cellulose columns	Gal:Glc:Ara = 43.39:51.43:5.18	1.14 × 10^4^	β-configurations	[67]
(35)	BGP-80	Hot water, purification on Sephadex G-75 and DEAE-Cellulose columns	Gal:Glc:Ara = 41.73:51.61:6.66	3.05 × 10^3^	α-Dominating configurations	[67]
(36)	GPS	Hot water	Glu:GalA:Gal:Ara:Rha:Man = 77.9:8.7:6.8:4.6:1.1:1.0			[27]
(37)	RG Polysaccharide	Hot water, purification by DEAE-cellulose column, Sepharose CL-6B column				[54]
(38)	GP II	Hot water, purification by DEAE-Sepharose CL-6B column		3 × 10^5^	60.06% (1→)- or (1→6)-glycosidic linkages and 39.94% (1→3)-glycosidic linkages	[68]
(39)	GP III	Hot water, purification by DEAE-Sepharose CL-6B column		4 × 10^5^	16.23% (1→)- or (1→6)-glycosidic linkages, 25.98% (1→2)-glycosidic linkages, and 57.79% (1→3)-glycosidic linkages	[68]
(40)	GP50-dHR	Hot water, purification by DEAE-Sepharose Fast Flow, dialysis (35 kDa), Sepharose CL-6B column		5.6 × 10^4^	HG backbone with hairy regions of RG-I	[70]
(41)	GP50-eHR	Hot water, purification by DEAE-Sepharose, dialysis (35 kDa), Sepharose CL-6B column		7.7 × 10^4^	HG backbone with hairy regions of RG-I	[70]
(42)	PGPW1	Hot water, purification by DEAE Sepharose Fast Flow, Sepharose 6 Fast Flow column	Glc:Gal:Man:Ara = 3.3:1.2:0.5:1.1	3.5 × 10^5^		[36]
(43)	WGFPA-1a	Hot water, purification by DEAE-Cellulose column, Sephacryl S-200 column	GalA:Rha:Gal:Ara:Glc:GlcA:Man = 3.9:3.1:47.2:39.4:2.1:4.0:0.3	1.6 × 10^4^		[34]
(44)	WGFPA-1b	Hot water, purification by DEAE-Cellulose column, Sephacryl S-200 column	GalA:Rha:Gal:Ara:Glc:GlcA:Man:Xyl:Fuc = 56.9:6.0:11.9:17.2:1.7:2.1:1.7:1.7:0.8	3.2 × 10^3^		[34]
(45)	WGFPA-2a	Hot water, purification by DEAE-Cellulose column, Sephacryl S-200 column	GalA:Rha:Gal:Ara:Glc:GlcA:Man = 14.3:9.0:35.3:39.1:0.8:1.3:0.3	8.6 × 10^4^		[34]
(46)	WGFPA-2b	Hot water, purification by DEAE-Cellulose column, Sephacryl S-200 column	GalA:Rha:Gal:Ara:Glc:GlcA:Xyl = 70.3:10.2:7.4:9.4:0.3:0.9:1.6	8.7 × 10^3^		[34]
(47)	WGFPA-3a	Hot water, purification by DEAE-Cellulose column, Sepharose CL-6B column	GalA:Rha:Gal:Ara:Glc:GlcA:Man:Xyl:Fuc = 19.0:13.4:27.5:35.9:0.9:0.7:0.9:1.6:0.1	3.0 × 10^5^		[34]
(48)	WGFPA-3b	Hot water, purification by DEAE-Cellulose column, Sepharose CL-6B column	GalA:Rha:Gal:Ara:Glc:GlcA:Xyl = 82.2:3.6:6.3:5.3:1.2:1.8:0.8	2.7 × 10^4^		[34]
(49)	GPW-1	Hot water, purification by DEAE-cellulose, Sephadex G-100 column	Fru:Rha:GalA:Glc:Gal:Ara = 0.85:1.40:6.35:3.57:26.33:61.75	8.51 × 10^5^	RG-I-rich	[52]
(50)	GPR-1	Hot water, purification by DEAE-cellulose, Sephadex G-100 column	Fru:Rha:GalA:Glc:Gal:Ara = 0.62:1.92:10.64:12.29:19.96:54.57	8.86 × 10^5^	RG-I-rich	[52]
(51)	GPS-1	Hot water, purification by DEAE-cellulose, Sephadex G-100 column	Fru:Rha:GalA:Glc:Gal:Ara = 2.71:2.12:11.27:13.31:39.92:30.67	9.61 × 10^5^	RG-I-rich	[52]
(52)	GPW-2	Hot water, purification by DEAE-cellulose, Sephadex G-100 column	Fru:Rha:GalA:Glc:Gal:Ara =6.48:3.64:29.12:6.19:22.33:32.24	2.95 × 10^5^	HG-rich	[52]
(53)	GPR-2	Hot water, purification by DEAE-cellulose, Sephadex G-100 column	Fru:Rha:GalA:Glc:Gal:Ara = 2.77:6.85:61.55:1.52:18.76:8.55	2.58 × 10^5^	HG-rich	[52]
(54)	GPS-2	Hot water, purification by DEAE-cellulose, Sephadex G-100 column	Fru:Rha:GalA:Glc:Gal:Ara = 5.57:6.71:68.09:9.36:6.62:3.65	3.39 × 10^5^	HG-rich	[52]
(55)	NGP-1a	50 mM Na_2_CO_3_ solution, purification by DEAE-cellulose column, Sepharose CL-6B	GalA:Rha:Gal:Ara = 6.3:4.3:58.8:30.6	1.23 × 10^5^	RG-I	[37]
(56)	NGP-2a	50 mM Na_2_CO_3_ solution, purification by DEAE-cellulose column, Sepharose CL-6B	GalA:Rha:Gal:Ara = 19.1:13.2:22.5:45.2	1.53 × 10^5^	RG-I	[37]
(57)	NGP-2b	50 mM Na_2_CO_3_ solution, purification by DEAE-cellulose column, Sepharose CL-6B	GalA:Rha:Gal:Ara = 8.7:7.1:60.3:23.9	4.9 × 10^3^	RG-I	[37]
(58)	1-KGP-P1	50 mM Na_2_CO_3_ solution, 1 M KOH solution, purification by Sepharose CL-6B	Glc:Xyl:Gal:Ara:GalA:Rha = 5.4:81.5:3.8:3.3:3.4:2.5	4.8 × 10^5^	Xylan	[37]
(59)	1-KGP-P2	50 mM Na_2_CO_3_ solution, 1 M KOH solution, purification by Sepharose CL-6B	Glc:Xyl:Gal:Ara:GalA:Rha = 44.1:19.3:8.0:19.6:3.6:5.4	2.7 × 10^3^	Xylan-xyloglucan	[37]
(60)	4-KGP-P1	50 mM Na_2_CO_3_ solution, 1 M KOH solution, purification by Sepharose CL-6B	Glc:Xyl:Gal:Ara:GalA:Rha = 4.7:37.6:7.9:8.7:27.9:13.7	4.46 × 10^5^	Xylan-RG-1	[37]
(61)	4-KGP-P2	50 mM Na_2_CO_3_ solution, 4 M KOH solution, purification by Sepharose CL-6B	Glc:Xyl:Gal:Ara:GalA = 39.1:26.9:17.7:8.3:1.9	1.23 × 10^5^	Xyloglucan	[37]
(62)	4-KGP-P3	50 mM Na_2_CO_3_ solution, 4 M KOH solution, purification by Sepharose CL-6B	Glc:Xyl:Gal:Ara = 56.8:12.9:10.6:19.7	2.6 × 10^3^	Xyloglucan	[37]
(63)	RG-CW-CP	Hot water, dialysis (12–14 kDa)	Ara:Fuc:Xyl:Rha:Man:Glc:Gal = 7.6:1.84:0.53:1.66:0.37:41.15:6.23:40.62			[41]
(64)	RG-HW-CP	Hot water, dialysis (12–14 kDa)	Ara:Fuc:Xyl:Rha:Glc:Gal = 7.26:1.15:0.14:1.64:52.84:5.49:31.48			[41]
(65)	RG-CW-EZ-CP	Hot water, dialysis (12–14 kDa), α-amylase and amyloglucosidase	Ara:Fuc:Rha:Man:Glc:Gal = 15.94:0.16:3.94:1.13:5.06:13.97:59.81			[41]
(66)	RG-HW-EZ-CP	Hot water, dialysis (12–14 kDa), α-amylase and amyloglucosidase	Ara:Fuc:Rha:Glc:Gal = 16.88:3.00:3.44:3.48:13.69:59.51			[41]
(67)	PG-F2	Hot water and RNase and DNase, purification by Q-Sepharose fast-flow column	Gal:Ara:Rha:GalA:GlcA:Xyl:Fuc = 1.6:1.8:2.3:76.6:16.9:0.1:0.6	1.2 × 10^4^		[74]
(68)	WGFPN	Hot water, purification by dialysis, DEAE-Cellulose column	Gal:Ara:Glc:Man = 78:14.3:5.2:2.5	1.1 × 10^4^	(1→4)-β-d-galactan and highly branched (1→6)-β-d-galactan, (1→6)-β-d-galactan, α-l-Araf through O-3	[73]
(69)	KRG-P	Hot water, purification by dialysis (20 kDa)	Glu:GalA:Gal:Ara:Rha = 60.5:19.7:11.0:6.8:1.9	1.06 × 10^5^		[72]
(70)	RGP-AP-I	Hot water, α-amylase, purification on a Sephadex G-100 column, dialysis (14 kDa)	Rha:Fuc:Ara:Xyl:Man:Gal:Glu:GalA:GluA = 9.5:0.1:35.0:0.3:0.4:30.4:1.7:18.4:1.5	9.6 × 10^4^	RG-I	[22]
(71)	RGP-AP-II	EtOH, α-amylase, purification on a Sephadex G-100 column, dialysis (14 kDa)	Rha:Fuc:Ara:Xyl:Man:Gal:Glu:GalA:GluA = 9.3:0.3:25.4:0.3:0.5:19.9:1.2:38.6:1.2			[22]
(72)	RGP-AP-III	EtOH, α-amylase, purification on a Sephadex G-100 column, dialysis (14 kDa)	Rha:Fuc:Ara:Xyl:Man:Gal:Glu:GalA:GluA = 6.8:0.8:19.6:0.3:0.5:24.6:2.6:38.9:1.4			[22]
(73)	RGP-AP-IV	EtOH, α-amylase, purification on a Sephadex G-100 column, dialysis (14 kDa)	Rha:Fuc:Ara:Xyl:Man:Gal:Glu:GalA:GluA = 1.4:0.5:4.2:0.3:0.5:10.5:4.1:74.4:0.7			[22]
(74)	FGWP	Hot water	Gal:Glc:Ara:Rha:Fuc = 9.16:60.59:10.11:1.81:0.14	6.5 × 10^6^		[40]
(75)	FGEP-C	Hot water, and cellulase	Gal:Glc:Ara:Rha:Fuc = 10.39:62.24:10.54:2.13:0.14			[40]
(76)	FGEP-A	Hot water, and α-amylase	Gal:Glc:Ara:Rha:Fuc = 12.6:50.86:10.89:1.76:0.17			[40]
(77)	FGEP-CA	Hot water, cellulase and α-amylase co-treatment	Gal:Glc:Ara:Rha:Fuc = 16.53:45.83:11.43:3.22:0.20	6.4 × 10^6^		[40]
(78)	EGP-N	Hot water, α-amylase and EDTA, purification by DEAE-Cellulose column	Gal:Ara:Glc = 3.9:4.6:88.7		Starch-like glucan, with minor AG	[42]
(79)	EGP-1a	Hot water, α-amylase and EDTA, purification by DEAE-Cellulose column, Sepharose CL-6B column	Glc = 100	4.5 × 10^5^	Starch-like glucan	[42]
(80)	EGP-1b	Hot water, α-amylase and EDTA, purification by DEAE-Cellulose column, Sepharose CL-6B column	GalA:Rha:Gal:Ara:Glc = 2.3:1.1:11.2:9.9:75.5	6.2 × 10^3^	More glucan with minor RG-I pectin	[42]
(81)	EGP-2a	Hot water, α-amylase and EDTA, purification by DEAE-Cellulose column, Sepharose CL-6B column	GalA:Rha:Gal:Ara:Glc = 32.7:8.0:27.6:27.7:3.4	4.2 × 10^5^	HG and RG-I pectin	[42]
(82)	EGP-2b	Hot water, α-amylase and EDTA, purification by DEAE-Cellulose column, Sepharose CL-6B column	GalA:Rha:Gal:Ara:Glc = 46.5:7.0:20.7:21.9:3.9	1.5 × 10^5^	HG and RG-I pectin	[42]
(83)	EGP-3a	Hot water, α-amylase and EDTA, purification by DEAE-Cellulose column, Sepharose CL-6B column	GalA:Rha:Gal:Ara:Glc = 52.8:8.7:16.9:17.7:3.9	4.3 × 10^5^	HG and RG-I pectin	[42]
(84)	EGP-3b	Hot water, α-amylase and EDTA, purification by DEAE-Cellulose column, Sepharose CL-6B column	GalA:Rha:Gal:Ara:Glc = 64.5:7.4:12.5:9.5:6.1	1.1 × 10^5^	HG and RG-I pectin	[42]
(85)	RG-I-3A-2	Enzyme-assisted, purification by DEAE-Cellulose, dialysis, Sepharose CL-6B column	Gal:Glc:Ara:Rha:Man:GalA:GlcA = 20.3:1.7:37.2:1.7:37.8:2.4	2.15 × 10^4^		[60]
(86)	RG-I-3A-4	Enzyme-assisted, purification by DEAE-Cellulose, dialysis, Sepharose CL-6B column	Gal:Glc:Ara:Rha:Man:GalA:GlcA = 14.8:1.9:39.6:0.9:40.2:2.5	7.2 × 10^3^		[60]
(87)	RG-I-3A-6	Enzyme-assisted, purification by DEAE-Cellulose, dialysis, Sepharose CL-6B column	Gal:Glc:Ara:Rha:Man:GalA:GlcA = 13.3:2.2:40.1:0.3:41.2:2.9	6.3 × 10^3^		[60]
(88)	RG-I-3A-16	Enzyme-assisted, purification by DEAE-Cellulose, dialysis, Sepharose CL-6B column	Gal:Rha:Man:GalA:GlcA = 5.7:44.8:1.9:45.6:1.9	6.0 × 10^3^	→4)-α-GalpA-(1→ and →2) -α-Rhap-(1→	[60]
(89)	RG-I-2	Enzyme-assisted, purification on a Sephadex G-25 column, dialysis	Gal:Ara:Rha:GalA = 12.4:14.5:1.7:44.3	4 × 10^3^	RG-I backbone with 1.5 side chains	[71]
(90)	RG-I-3B	Enzyme-assisted, purification on a Sephadex G-25 column, dialysis	Gal:Ara:Rha:GalA = 13.4:11.9:14.1:44.6	6 × 10^3^	RG-I backbone with 4 side chains	[71]
(91)	RG-I-4	Enzyme-assisted, purification on a Sephadex G-25 column, dialysis	Gal:Ara:Rha:GalA = 19.5:9.2:21.8:33.8	6 × 10^4^	RG-I backbone, Ara, and Gal side chains	[71]
(92)	WGPE-N	4000 U α-amylase, dialysis (3.5 kDa), purification by DEAE-Cellulose	Gal:Glc:Ara = 2.3:94.8:2.9		Starch-like glucans	[39]
(93)	WGPE-1a	4000 U α-amylase, dialysis (3.5 kDa), purification by DEAE-Cellulose, Sephadex G-75 column	Gal:Glc:Ara:Rha = 30.9:44.3:21.9:2.1	1.1 × 10^4^	AG chains	[39]
(94)	WGPE-1b	4000 U α-amylase, dialysis (3.5 kDa), purification by DEAE-Cellulose, Sephadex G-75 column	Gal:Glc:Ara:Rha:GalA = 8.3:72.9:0.8:1.9:2.8	5.5 × 10^3^	More glucan and less AG	[39]
(95)	WGPE-2a	4000 U α-amylase, dialysis (3.5 kDa), purification by DEAE-Cellulose, Sepharose CL-6B column	Gal:Glc:Ara:Rha:GalA =36.5:1.2:39.7:8.3:13.7	4.3 × 10^6^	RG-I pectin branched with α→1,5/1,3,5-arabinan and β-1,4-galactan side chains	[39]
(96)	WGPE-2b	4000 U α-amylase, dialysis (3.5 kDa), purification by DEAE-Cellulose, Sepharose CL-6B column	Gal:Glc:Ara:Rha:GalA = 14.1:2.9:16.1:4.6:62.2	1.2 × 10^5^	HG pectin with 4-*O*-methyl-α-d- GalA	[39]
(97)	WGPE-3a	4000 U α-amylase, dialysis (3.5 kDa), purification by DEAE-Cellulose, Sepharose CL-6B column	Gal:Ara:Rha:GalA =23.0:30.7:11.4:34.9	4.2 × 10^6^	RG-I pectin branched with α- 1,5/1,3,5-arabinan and β-1,4-galactan side chains	[39]
(98)	WGPE-3b	4000 U α-amylase, dialysis (3.5 kDa), purification by DEAE-Cellulose, Sepharose CL-6B column	Gal:Glc:Ara:Rha:GalA =5.2:4.1:5.6:3.4:81.7	5.0 × 10^4^	HG pectin with 4-*O*-methyl-α-d-GalA	[39]
(99)	WGPA-P2A	Hot water, hydrolyzed by Endo-PG, purification by Sephadex G-25, DEAE-Sepharose Fast Flow, Sephadex G-75	Gal:Glc:Ara:Rha:Man:GalA:GlcA = 41.1:1.7:30.1:11.5:1.3:12.2:2.1	7.8 × 10^4^	RG-I	[75]
(100)	WGPA-P2B	Hot water, hydrolyzed by Endo-PG, purification by Sephadex G-25, DEAE-Sepharose Fast Flow, Sephadex G-75	Gal:Glc:Ara:Rha:Man:GalA:GlcA = 11.4:4:20.3:13.4:0.6:47.9:2.4	3.7 × 10^3^	RG-II	[75]
(101)	RG-I-1	Hot water, hydrolyzed by Endo-PG, purification by Sephadex G-25 column, DEAE-Sepharose Fast Flow column, Sephadex G-75	GalA:Rha:Gal:Ara:Man:Glc:GlcA:Fuc = 26.8:12.8:21.2:13.0:5.9:7.2:7.4:2.0	5 × 10^3^		[76]
(102)	RG-I-2	Hot water, hydrolyzed by Endo-PG, purification by Sephadex G-25 column, DEAE-Sepharose Fast Flow column, Sephadex G-75	GalA:Rha:Gal:Ara:Man:Glc:GlcA:Fuc = 44.3:11.7:12.4:14.5:1.0:4.4:5.8:3.8	4 × 10^3^	4-*O*-Me-b-d-GlcAp at the non-reducing terminals of branched chains	[76]
(103)	RG-I-3A	Hot water, hydrolyzed by Endo-PG, purification by Sephadex G-25 column, DEAE-Sepharose Fast Flow column, Sepharose CL-6B	GalA:Rha:Gal:Ara:Man:Glc:GlcA:Fuc = 32.2:11.1:31.6:16.3:2.1:1.9:3.0:0.7	4.5 × 10^4^	AG-I	[76]
(104)	RG-I-3B	Hot water, hydrolyzed by Endo-PG, purification by Sephadex G-25 column, DEAE-Sepharose Fast Flow column, Sepharose CL-6B	GalA:Rha:Gal:Ara:Man:Glc:GlcA:Fuc = 44.6:14.1:13.7:11.9:1.3:2.5:3.7:2.3	6 × 10^3^	AG-I	[76]
(105)	RG-I-4	Hot water, hydrolyzed by Endo-PG, purification by Sephadex G-25 column, DEAE-Sepharose Fast Flow column, Sepharose CL-6B	GalA:Rha:Gal:Ara:Man:Glc:GlcA:Fuc = 33.8:21.8:19.5:9.2:0.4:3.0:2.2:1.5	6 × 10^4^	AG-I	[76]
(106)	MPPG	Microwave, purification by DEAE-Cellulose column	Gal:Glu:Rha:Man:GalA:Rib:GluA:Arab = 2.7:141.42:1.85:3.94:1.43:4.55:1:5.15	3.69 × 10^6^		[45]
(107)	G1	Microwave assisted purification (1100 W), separation by ultrafiltration (3 kDa)	Gal:Glc:Ara:Rha:Man:GalA = 4.37:3.95:5.16:0.37:0.17:3.9	3.5 × 10^3^–1.1 × 10^5^		[61]
(108)	G2	Microwave assisted purification (1100 W), separation by ultrafiltration (3 kDa)	Gal:Glc:Ara:Rha:Man:GalA = 4.53:4.12:5.03:0.79:0.57:3.85			[61]
(109)	G3	Microwave assisted purification (1100 W), separation by ultrafiltration (3 kDa)	Gal:Glc:Ara:Rha:Man:GalA = 4.33:4.02:5.11:0.75:0.95:4.06			[61]
(110)	G4	Microwave assisted purification (1100 W), separation by ultrafiltration (3 kDa)	Gal:Glc:Ara:Rha:Man:GalA = 4.62:4.34:5.1:0.65:0.65:3.9			[61]
(111)	G5	Microwave assisted purification (1100 W), separation by ultrafiltration (3 kDa)	Gal:Glc:Ara:Rha:Man:GalA = 4.62:4.46:5.35:0.71:0.92:4.47			[61]
(112)	PPG	Ultrasonication extract, dialysis (3.5 kDa)	Ara:Rha:GalA:Man:Glc:Gal = 28:3:1:4:70:44	1.499 × 10^6^, 5.335 × 10^4^	1→4 glycosidic linkages as linear backbone	[30]

**Table 2:** Structure and composition of American ginseng polysaccharides.

No.	Polysaccharide Name	Extraction and Purification	Composition Ratio of Sugars (Mass Percentage or Molar Ratio)	Molecular Weight (Da)	Structure Feature	Refs
(1)	WPS-1	Hot water, purification on DEAE-Sepharose CL-6B and Sepharose CL-6B	Gal:Glc:Ara:Rha:Man = 18.7:55.2:21.2:2.3:2.6	1.54 × 10^6^	(1→6)-α-d-Glcp, (1→5)-α-l-Araf and (1→4)- β-d-Rhap	[57]
(2)	WPS-2	Hot water, purification on DEAE-Sepharose CL-6B and Sepharose CL-6B	Gal:Glc:Ara:Rha:Man = 20.7:46.8:27.9:1.7:2.9	1.41 × 10^4^	(1→6)-α-d-Glcp and (1→5)-α-l-Araf	[57]
(3)	SPS-1	Hot water, purification on DEAE-Sepharose CL-6B and Sepharose CL-6B	Gal:Glc:Ara:Man:GalA:GlcA:Xyl = 28.6:15.9:22.3:9.2:13.6:3.5:6.9	3.62 ×10^5^	(1→6)-α-d-Glcp, (1→4)-α-d-Manp, (1→5)-α-l-Araf, β-d-Galp and β-d-xylose	[57]
(4)	SPS-2	Hot water, purification on DEAE-Sepharose CL-6B and Sepharose CL-6B	Gal:Glc:Ara:Man:GalA:GlcA:Xyl = 22.5:25.3:14.2:7.9:16.9:7.9:5.3	9.70 × 10^6^	(1→6)-α-d-Glcp, (1→4)-α-d-Manp, (1→5)-α-l-Araf, β-d-Galp, β-d-xylose and O-acetyl group	[57]
(5)	SPS-3	Hot water, purification on DEAE-Sepharose CL-6B and Sepharose CL-6B	Gal:Glc:Ara:Rha:Man:GalA:Xyl = 15.2:11.5:19.2:2.1:12:26.3:9.6	5.12 × 10^5^	(1→6)-α-d-Glcp, (1→4)-α-d-Manp, (1→5)-α-l-Araf, β-d-Galp, β-d-xylose, O-acetyl group and (1→4)-β-d-Rhap	[57]
(6)	GSP	Hot water, purification by dialysis (12 kDa)	Rha:Ara:Gal:Glc = 1:4:8:8	8.5 × 10^4^	The main sugar residues were 4-α-d-GalpA and 4-α-d-GalpA, other residues such as 2-α-l-Rhap, 2,4-α-l-Rhap, α-l-Araf, β-d-Galp, 4-β-d-Galp	[77]
(7)	quinquefolans A	Hot water, purification by DEAE-Toyopearl, Sephacryl S-200 and S-50	Glc:Man = 2.3:1	>2 × 10^3^		[83]
(8)	quinquefolans B	Hot water, purification by DEAE-Toyopearl, Sephacryl S-200 and S-50	Glc:Man = 5.5:1	>2 × 10^3^		[83]
(9)	quinquefolans C	Hot water, purification by DEAE-Toyopearl, Sephacryl S-200 and S-50	Xyl = 1	>2 × 10^3^		[83]
(10)	PPQN	Hot water, purification by DEAE Sepharose Fast Flow column	Gal:Glc = 1.15:1	3.1×10^3^		[80]
(11)	PPQA2	Hot water, purification by DEAE Sepharose and Sephacryl S-300	Gal:Glc:Ara:Rha:Man:GalA:GlcA = 7.2:12.5:8:4:2.9:26.6:38.8	2.3 ×10^4^	O-acetyl groups and β-arabinopyranosyl residue	[51]
(12)	PPQA4	Hot water, purification by DEAE Sepharose and Sephacryl S-300	Gal:Glc:Ara:Rha:Man:GlcA = 23.9:41.3:19.7:5.1:8.1:2	1.2 × 10^5^	β-arabinopyranosyl residue	[51]
(13)	PPQA5	Hot water, purification by DEAE Sepharose and Sephacryl S-300	Gal:Glc:Ara:Rha:Man:GalA:GlcA = 10.8:32.4:8.5:3.2:5.3:15.5:24.4	5.3×10^3^	O-acetyl groups and β-arabinopyranosyl residue	[51]
(14)	AGC1	Hot water, purification by DEAE-Sepharose column	Gal:Glc:Ara:Xyl:Glu:Rha:Man = 60:6.29:19.2:11.4:1.5:0.8	5.2 × 10^3^	3-Galp (48.5%), 3,6-Galp (10.2%), t-Galp (5.2%), 6-Galp (4.4%), 4-Glcp (5.7%), 4-Arap/5-Araf (4.0%) and t-Araf (4.5%)	[78]
(15)	AGC3	Hot water, purification by DEAE-Sepharose column	Gal:Glc:Ara:Rha:Man:GlcA = 74.3:92:7.8:8.1:6.8:1	4.81 × 10^3^, 3.2 ×10^4^	RG-I pectin, t-Galp (47.7%), 4-Galp (15.6%), 2,4-Rhap (8.1%), 6-Galp (8.1%) and 4-GalAp (6.8%)	[79]
(16)	PPQ	Hot water, purification by DEAE Sepharose Fast Flow column, Sephacryl S-200 High Resolution column	Glc:Gal = 2.1:1	5.4 × 10^4^		[81]
(17)	GPS	Hot water		1.092 × 10^6^		[82]
(18)	AEP-1	0.3 M NaOH, purification by DEAE Sepharose Fast Flow column, Sephacryl S-300 High Resolution column	Gal:Glc:GalA = 0.97:4.67:3.92		d-GalpA	[38]
(19)	AEP-2	0.3 M NaOH, purification by DEAE Sepharose Fast Flow column, Sephacryl S-300 High Resolution column	Gal:Glc:Ara:Man:GalA = 1.68:3.02:1.03:0.76:3.65			[38]
(20)	Q1	Microwave assisted purification (1.1 kW), separation by ultrafiltration (3 kDa)	Rha:Ara:GalA:Man:Glc:Gal = 0.39:3.06:2.16:0.14:2.95:2.36:11.1	8.54 × 10^4^		[61]
(21)	Q2	Microwave assisted purification (1.1 kW), separation by ultrafiltration (3 kDa)	Rha:Ara:GalA:Man:Glc:Gal = 0.46:3.16:2.29:0.13:3.13:2.47:11.6			[61]
(22)	Q3	Microwave assisted purification (1.1 kW), separation by ultrafiltration (3 kDa)	Rha:Ara:GalA:Man:Glc:Gal = 0.48:3.18:2.10:0.09:3.02:2.47:11.3			[61]
(23)	Q4	Microwave assisted purification (1.1 kW), separation by ultrafiltration (3 kDa)	Rha:Ara:GalA:Man:Glc:Gal = 0.45:3.23:2.19:0.09:2.97:2.40:11.3			[61]
(24)	Q5	Microwave assisted purification (1.1 kW), separation by ultrafiltration (3 kDa)	Rha:Ara:GalA:Man:Glc:Gal = 0.49:3.09:2.12:0.10:3.06:2.43:11.3			[61]
(25)	PPQ	Ultrasonication extract, dialysis (3.5 kDa)	Ara:Rha:GalA:Man:Glc:Gal = 31:4:1:2:72:59	3.626 × 10^6^, 2.520 × 10^5^, 5.356 × 10^4^	1→4 glycosidic linkages as linear backbone	[30]

**Table 3:** Structure and composition of notoginseng polysaccharides.

No.	Polysaccharide Name	Extraction and Purification	Composition Ratio of Sugars (Mass Percentage or Molar Ratio)	Molecular Weight (Da)	Structure Feature	Refs
(1)	Sanchinan-A	MeOH and hot water, purification by Sephadex G-50 column		1.5 × 10^6^	β-d-(1→3)-linked galactopyranosyl residues	[84]
(2)	PF3111	Hot water, purification by Sephadex G-50 column, Sephadex-DEAE A-50 column twice	Gal:Glc:Ara:Man = 3.5:10.8:1:3.5	6.85 × 10^5^		[56]
(3)	PF3111	Hot water, purification by Sephadex G-50 column, Sephadex-DEAE A-50 column twice	Gal:Glc:Ara:Man = 2.9:5.3:1:2.8	3.7 × 10^4^		[56]
(4)	PBGA11	0.01 M NaOH, purification by Sephadex G-50 column, Sephadex-DEAE A-50 column twice	Gal:Glc:Ara:Man = 3.1:4.2:1:5.3	4.5 × 10^4^		[56]
(5)	PBGA12	0.01 M NaOH, purification by Sephadex G-50 column, Sephadex-DEAE A-50 column twice	Gal:Glc:Ara:Man = 2.5:7.2:1:4.3	7.6 × 10^5^		[56]
(6)	AIR	Sonicated with MeOH	Gal:Glc:Ara:GalA = 11:75:3:11		4-Glcp (72 mol%)	[50]
(7)	Fr_1MKOH_	1 M KOH, DMSO, dialysis (3.5 kDa)			4-galactan, heteroxylan, starch; 2,4-Rhap, 4-Xylp, 4-Galp, terminal Glcp, 4-GalAp	[58]
(8)	1MD3-G2	1 M KOH, DMSO, dialysis (3.5 kDa), DEAE-Sepharose CL-6B column		1.14 × 10^6^	Glucuronoarabinoxylan, HG, RG-I, 4-galactan, arabinan	[58]
(9)	Arabinoglucogalactan (1)	Hot water, purification by DEAE- cellulose column twice, Sephadex G-100 column, Sephacryl S-300 column	Ara:Glu:Gal = 1:1:8	6.7 × 10^4^	a backbone of (1→3)-linked β-d-galactofuranosyl residues, with branches of α-l-Araf-(1→4)-β-d-Glcp-(1→residues at O-6	[85]
(10)	MRP	Hot water	Gal:Glc:Ara:Rha:Man = 16.9:28:3.2:4.2:47.4			[26]
(11)	BRP	Hot water	Gal:Glc:Ara:Man = 26.9:17.9:1.7:10.4			[26]
(12)	FRP	Hot water	Gal:Glc:Ara:Man = 10.3:49.3:1.7:22.2			[26]
(13)	RN1	Hot water, purification by DEAE-cellulose column	Gal:Ara = 43.7:56.3	2.1 × 104	A backbone of 1,6-linked Galp branched at C3 by side 1,3-linked Galp, with branches attached at position O-3,1,5-linked, 1,3,5 linked, terminal Ara and terminal Gal	[86]
(14)	N1	Microwave assisted purification (1.1 kW), separation by ultrafiltration (3 kDa)	Rha:Ara:GalA:Man:Glc:Gal = 0.14:2.83:0.63:0.17:7.28:2.67:13.7			[61]
(15)	N2	Microwave assisted purification (1.1 kW), separation by ultrafiltration (3 kDa)	Rha:Ara:GalA:Man:Glc:Gal = 0.11:2.83:0.56:0.13:7.64:2.64:13.9			[61]
(16)	N3	Microwave assisted purification (1.1 kW), separation by ultrafiltration (3 kDa)	Rha:Ara:GalA:Man:Glc:Gal = 0.14:3.03:0.47:0.23:7.72:2.69:14.3			[61]
(17)	N4	Microwave assisted purification (1.1 kW), separation by ultrafiltration (3 kDa)	Rha:Ara:GalA:Man:Glc:Gal = 0.12:3.03:0.40:0.17:7.54:2.64:13.9			[61]
(18)	N5	Microwave assisted purification (1.1 kW), separation by ultrafiltration (3 kDa)	Rha:Ara:GalA:Man:Glc:Gal = 0.14:3.02:0.57:0.13:8.25:2.84:14.9			[61]
(19)	MRP5A	Hot water, purification by DEAE-52 Cellulosee, Sephadex G-100 column	Gal:Glu:Ara:Rha = 15.9:69.8:6.6:7.7	1.138 × 10^5^		[87]
(20)	MRP5	Hot water, purification by DEAE-52 Cellulosee, Sephadex G-100 column	Gal:Glu:Ara:Rha = 29.7:51.2:14.5:3.6	9.16 × 10^4^	→3)-β-Glcp-(1→, →3)-β-Galp-(1→, →3, 6)- β-Galp-(1→, →3)-β-Galp-(1→, →3, 6)-β-Galp-(1→, →3)-α- Rhap-(1→, →3)-α-Araf-(1→, and α-Araf-(1→ residues	[87]
(21)	PNPS-0.5M	Hot water, purification by DEAE-FAST-FLOW Cellulose column	Glc:Ara:Rha:Man = 22.5:4.5:3.8:0.2	2.6 × 10^6^	(1→)-linked Araf, (1→) linked Rhap, (5→1)-linked Araf, (4→1)-linked GalA, and (3,6→1)-linked Galp	[43]
(22)	PNPS-0.3	Hot water, purification by DEAE-52 cellulose, Sephadex G-50	Gal:Glc:Ara:Rha:GalA = 33.3:4.5:25.2:15.5:17.1	7.6655 × 10^4^	A backbone of →4)-α-d-GalAp-(1→4-β-l-Rhap-1→4)-β-d-Galp-(1→residues, with an α-l-Araf-1→5)-α-l-Araf-(1→branch connecting to the backbone at O-3 of →4-β-l-Rhap-1→	[32]
(23)	PNPN	Hot water, purification by DEAE-Cellulose column, Sepharose CL-6B column	Gal:Glc:Ara:Rha:Man = 12.3:82.9:2.3:1.3:1.2			[29]
(24)	PNPA-1A	Hot water, purification by DEAE-Cellulose column, Sepharose CL-6B column	Gal:Glc:Ara:Rha:Man:GalA = 63.2:2.4:27.7:0.8:0.9:5	8.8 × 10^4^	1,4-β-d-galactan and 1,5-α-l-arabinan, small amounts of AG-II, and minor RG-I domains	[29]
(25)	PNPA-2A	Hot water, purification by DEAE-Cellulose column, Sepharose CL-6B column	Gal:Ara:Rha:Man:GalA:GlcA = 46:33.4:6:2:11.6:1	1.01 × 10^5^	1,4-β-d-galactan and 1,5-α-l-arabinan, small amounts of AG-II, and minor RG-I domains	[29]
(26)	PNPA-3A	Hot water, purification by DEAE-Cellulose column, Sepharose CL-6B column	Gal:Glc:Ara:Rha:Man:GalA = 32.7:2.2:28.3:15.5:4:15.9	2.70 × 10^5^	Typical RG-I type pectin with 1,4-β-d-galactan and 1,5/1,3,5-α-l-arabinan side chains	[29]
(27)	PNPA-1B	Hot water, purification by DEAE-Cellulose column, Sepharose CL-6B column	Gal:Glc:Ara:Rha:Man:GalA:GlcA = 29.3:4.5:10.4:9.6:2.9:40.6:0.6	3 × 10^3^	HG, RG-I and RG-II	[29]
(28)	PNPA-2B	Hot water, purification by DEAE-Cellulose column, Sepharose CL-6B column	Gal:Ara:Rha:Man:GalA:GlcA = 8.3:8.2:7.5:0.8:74.4:0.8	6 × 10^3^	HG, RG-I and RG-II	[29]
(29)	PNPA-3B	Hot water, purification by DEAE-Cellulose column, Sepharose CL-6B column	Gal:Glc:Ara:Rha:Man:GalA:GlcA = 8.8:1.6:5.1:5.2:1.4:75.8:0.9	1.8 × 10^4^	HG, RG-I and RG-II	[29]
(30)	PPN	Ultrasonication extract, dialysis (3.5 kDa)	Ara:GalA:Man:Glc:Gal = 2:1:2:83:7	6.014 × 10^5^, 5.065 × 10^4^	1→4 glycosidic linkages as linear backbone	[30]
(31)	NPPN	Hot water, purification by DEAE Sepharose-Fast Flow column, dialysis		1.81 × 10^4^		[88]
(32)	APPN I	Hot water, purification by DEAE Sepharose-Fast Flow column, dialysis		7.047 × 10^4^		[88]
(33)	APPN II	Hot water, purification by DEAE Sepharose-Fast Flow column, dialysis		1.056 × 10^5^		[88]
(34)	APPN III	Hot water, purification by DEAE Sepharose-Fast Flow column, dialysis		9.233 × 10^4^		[88]
(35)	APPN IV	Hot water, purification by DEAE Sepharose-Fast Flow column, dialysis		9.017 × 10^4^		[88]
(36)	FPNP	Hot water, purification by DEAE- Sepharose Fast Flow				[2]

**Table 4:** Structure of ginseng, American ginseng and notoginseng polysaccharides.

Polysaccharides	Extraction Method	Purification	M.W. (kDa)	Monosaccharide Compositions	Structure Features
ginseng	Hot water	DEAE-Cellulose Sepharose CL-6B Sephadex G-25 Sephadex G-50 Sephadex G-75 Sephadex G-100 Sephacryl S-200 Dialysis (3.5 kDa) Ultrafiltration (3 kDa)	0.1–2070	Ara, Arab, Fru, Fuc, Gal, GalA, Glc, GlcA, Man, Rha, Rib, Xyl	AG-I, AG-II, HG, RG-I, RG-II, and starch-like glucans
	Alkaline		2.6–480	Ara, Gal, GalA, Glc, Xyl	RG-I, amyloses, xyloglucan, and starch-like glucan
	Enzyme		4–4300	Ara, Fuc, Gal, GalA, Glc, GlcA, Man, Rha, Xyl	AG-I, AG, HG, RG-I, and starch-like glucans
	EDTA		6.2–450	Ara, Gal, Glc, GalA, Rha	AG, HG, RG-I, and starch-like glucan
	Microwave Ultrasonication		3.5–3690 53.35–1499	Ara, Arab, Gal, GalA, Glc, GlcA, Man, Rha, Rib Ara, Gal, GalA, Glc, Man, Rha	1→4 glycosidic linkages as linear backbone
American ginseng	Hot water	DEAE Sepharose Fast Flow Sephacryl S-200 Sephacryl S-300 Sepharose CL-6B Ultrafiltration (3 kDa) Dialysis (3.5 kDa)	3.1–9700	Ara, Gal, GalA, Glc, GlcA, Man, Rha, Xyl	RG-I, (1→6)-α-d-Glcp, (1→4)-α-d-Manp, (1→5)-α-l-Araf, β→-Galp, β-d-xylose, O-acetyl group, β-arabinopyranosyl residue
	Alkaline			Ara, Gal, GalA, Glc, Man	d-GalpA
	Microwave		85.4	Ara, Gal, GalA, Glc, Man, Rha	
	Ultrasonication		53.56–3626	Ara, Gal, GalA, Glc, Man, Rha	1→4 glycosidic linkages as linear backbone
notoginseng	Hot water	Sephadex G-50 Sephadex-DEAE A-50 DEAE-Sepharose CL-6B Ultrafiltration (3 kDa) Dialysis (3.5 kDa)	3–2600	Ara, Gal, GalA, Glc, GlcA, Man, Rha	AG-II, HG, RG-I, RG-II, (4→1)-linked GalA, 1,4-β-d-galactan and 1,5-α-l-arabinan
	Alkaline		45–1140	Ara, Gal, Glc, Man	HG, RG-I, glucuronoarabinoxylan, 4-galactan, arabinan, heteroxylan, starch; 2,4-Rhap, 4-Xylp, 4-Galp, terminal Glcp, 4-GalAp
	MeOH		45–1500	Ara, Gal, GalA, Glc	β-d-(1→3)-linked galactopyranosyl residues, 4-Glcp
	Microwave			Ara, Gal, GalA, Glc, Man, Rha,	
	Ultrasonication		50.65–601.4	Ara, Gal, GalA, Glc, Man	1→4 glycosidic linkages as linear backbone

**Table 5:** Activities and molecular mechanisms of polysaccharides from ginseng, American ginseng, and notoginseng.

Activities/Polysaccharides		Models	Molecular Mechanisms	Refs
**Anti-cancer effect** ***Ginseng polysaccharides***				
(1)	GPS	Mice macrophage, K562, HL-60, or KG1 cells	Possesses a potent antitumor activity by stimulating TNF-α, IL-1, IL-6, and NO production in macrophage against leukemia	[89]
(2)	GPS	MDA-MB-231 breast cancer cells	Activates p65-IKZF1 signaling and apoptosis to inhibit cell proliferation	[90]
(3)	GBPE, GBPP	HCT-116 and HT-29 human colon cancer cells and CD4^+^ IFN-γ^+^ cells	Inhibits IL-8 secretion and CD4^+^ IFN-γ^+^ cell (Th1) and CD4^+^ FoxP3^+^ cell (Treg) differentiation to suppress cancer cell growth	[91]
(4)	GBPP	B16-BL6 melanoma xenograft mice model	Increases IL-6, IL-12, TNF-α, IFN-γ, and granzyme B of NK cells to suppress tumor colonies	[18]
(5)	NFP	B16F10 melanoma xenograft mice model	Inhibits melanoma cell metastasis to the lung by enhancing immunity	[92]
(6)	GS-P	Colon 26-M3 cells and xenograft mice model	Promotes the macrophages and NK cells to exhibit anti-metastatic activity	[93]
(7)	WGPN	S 180 tumor-bearing mice	Stimulates lymphocytes proliferation and macrophage activity and mitigates the damage of immune system induced by 5-fluorouracil	[94]
(8)	WGPA, WGPA-3-HG	HT-29 colon cancer cells	Inhibits cell proliferation and cell cycle arrest in the G_2_/M phase	[95]
(9)	MWGPA-3-HG	HT-29 colon cancer cells	Increases the percentages of S and G_2_/M phase cells and induces apoptosis by activating caspase-3	[95]
(10)	PGPW1	HGC-27 gastric cancer cells	Decreases migration and invasion by regulation of Twist, AKR1C2 and NF1 to mediate epithelial–mesenchymal transition	[36]
(11)	GP	Lewis lung carcinoma xenograft mice model	Increases the ratio of CD4^+^/CD8^+^ T lymphocyte in peripheral blood and promotes NK cytolytic activity	[97]
(12)	GFP1	Lewis lung carcinoma xenograft mice model	Promotes ConA or LPS-induced spleen lymphocytes proliferation and elevates NK cell activity and the ratio of CD4^+^/CD8^+^ to inhibit tumor growth and metastasis	[96]
(13)	GPS	96 patients with non-small cell lung cancer	Increases Th1 cytokines (INF-γ, IL-2) and decreases Th2 cytokines (IL-4, IL-5)	[98]
(14)	GPs	Lewis lung carcinoma xenograft mice model	Alters gut microbiota and kynurenine/tryptophan ratio to potentiate antitumor effect of anti-PD-1/PD-L1 immunotherapy	[99]
***American ginseng polysaccharides***				
(1)	GPS NPs	UVB-induced SKH1 hairless mice	Inhibits the pro-inflammatory cytokine levels, epidermal proliferation, and skin cancer	[82]
***Notoginseng polysaccharides***				
(1)	RN1	HMEC-1 microvascular endothelial cells, BxPC3 pancreatic cancer xenograft mice model	Inhibits BMP2/Smad1/5/8/Id1 signaling to inhibit tumor angiogenesis	[86]
(2)	PPN	H22 cells and tumor-bearing mice	Activates CD4^+^ T-cells and elevates serum IL-2 to inhibit tumor growth and prolong the survival	[100]
(3)	NPPN	H22 cells and tumor-bearing mice	Inhibits tumor growth via myelosuppression after combination with CTX	[88]
**Immunomodulatory effect**				
***Ginseng polysaccharides***				
(1)	FGWP-CA	CTX-induced immunosuppressed mice	Increases spleen and thymus indices, lymphocyte proliferation, NK cell activity, leukocyte counts, and IL-6, IL-12, and TNF-α levels	[40]
(2)	GPS	CTX-induced mice	Increases NK cell number and upregulates the levels of perforin and granzyme to activate NK cells	[101]
(3)	APG	Autoimmune encephalomyelitis mice	Promotes Treg cell generation through Foxp3 activation and inhibits the production of inflammatory cytokines, including IFN-γ, IL-1β, and IL-17	[102]
(4)	APG	Autoimmune encephalomyelitis-induced SJL/J mice	Modulates the infiltration of CD4^+^ T cells and CD11b^+^ macrophages into the spinal cord and decreases the amounts of IFN-γ, IL-17, and TNF-α	[103]
(5)	GPNE-I	ConA or LPS-induced lymphocyte cells	Increases T and B lymphocyte proliferation	[20]
(6)	WGPA-2-RG	Normal mice spleen lymphocytes and LPS-induced peritoneal macrophages	Decreases lymphocyte proliferation and macrophage nitrite production and slightly increases macrophage phagocytosis	[64]
(7)	Ginsan	Dendritic cells	Increases the levels of IL-16 and TNF-α and the expression of CD86	[23]
(8)	PS-NPs	RAW 264.7 macrophages	Increases the levels of TNF-α, IL-1β, and IL-6 and NO production	[104]
(9)	RGAP	RAW 264.7 macrophages	Enhances NO production and increases nuclear transcription factors by activating the ERK/JNK and NF-κB/AP-1 signaling pathways	[49]
(10)	RGAP	BALB/c mice spleen cells	Promotes spleen cell proliferation and NO production	[105]
(11)	RGAP	BALB/c mice spleen cells and intraperitoneal macrophages	Increases the numbers of IgM antibody-forming cells, T cells, B cells, and macrophages	[106]
(12)	RGAP	Immunosuppressed mice	Increases plaque-forming cells in the spleen in response to LPS and sheep red blood cells	[107]
(13)	RGP-AP-I	BALB/c mic peritoneal macrophages	Augments the production of IL-6, IL-12, and TNF-α	[22]
(14)	RG-CW-EZ-CP	BALB/c mice intraperitoneal macrophages and Peyer’s patch cells from small intestines of C3H/He mice	Enhances intestinal immune system and macrophage activity by upregulating the phosphorylation of ERK, JNK, and p38	[41]
(15)	NGP	Murine bone marrow dendritic cells	Increases the expressions of MHC II, CD80, CD86, CD83, and CD40 and decreases TNF-α levels	[108]
(16)	PGP-SL	Murine spleen lymphocytes	Regulates the Ca^2+^/calcineurin/NFAT/IL-2 signaling pathway	[109]
(17)	GMP	Murine peritoneal macrophages	Increases the production of reactive oxygen intermediates	[110]
(18)	Gps	LPS-induced weaned piglets	Regulates the TLR4/MyD88-NF-κB pathway to reduce immunological stress	[111]
***American ginseng polysaccharides***				
(1)	High molecular weight polysaccharides	Human peripheral blood mononuclear cells	Triggers the ERK, PI3K, p38, and NF-κB signaling pathways	[112]
(2)	AGRPS	Rat alveolar macrophage	Increases NO production and TNF-α concentration	[113]
(3)	WPS-1, WPS-2, SPS-1, SPS-2, SPS-3	Mice lymphocytes and macrophages	Increases macrophage phagocytosis, NO production, and splenic lymphocyte proliferation	[57]
(4)	AGP	CTX-induced mice	Enhances CD4^+^ T cells and IgA-secreting cells and regulates gut microbiota	[114]
(5)	AGP	RAW 264.7 macrophages	Up-regulates the production of NO and cytokines	[115]
(6)	AGC1	RAW 264.7 macrophages and primary murine splenocytes	Enhances NO, TNF-α, IL-6, MCP-1, and GM-CSF levels to increase cell proliferation via the NF-κB signaling pathway	[78]
(7)	AGC3	RAW 264.7 macrophages and primary murine splenocytes	Enhances IL-6, TNF-α, GM-CSF, and MCP-1 levels via the NF-κB (p65/RelA) and p38 signaling pathways	[79]
(8)	PPQA2, PPQA4, PPQA5	RAW 264.7 macrophages	Increases NO, TNF-α, and IL-6 production	[51]
(9)	PPQN	RAW 264.7 macrophages	Inhibits the production of TNF-α, IL-1β, and IL-6	[80]
(10)	GPS NPs	RAW 264.7 macrophages and Swiss albino mice	Increases NO, TNF-α, IL-1β, and IL-6	[116]
***Notoginseng polysaccharides***				
(1)	PBGA12	Mice peritoneal macrophages	Enhances IFN-γ and TNF-α to stimulate complement system	[56]
(2)	Fr1MKOH	Human polymorphonuclear neutrophils	Shows complement-fixing activity and mitogenic effect through regulation of ROS and IFN-γ	[58]
(3)	PNPS-0.3	Bone marrow dendritic cells	Increases the amounts of TNF-α and IL-12 and induces T-cell immune response (CD4, CD8, CD69, and MHC II) by triggering the TLR4/TLR2-NF-κB signaling pathway	[32]
**Anti-oxidative activity**				
***Ginseng polysaccharides***				
(1)	Native ginseng polysaccharides	In vitro	Scavenges DPPH, hydroxyl, or superoxide anion radicals	[24]
(2)	Ginseng polysaccharides	In vitro	Scavenges DPPH free radicals	[25]
(3)	Ginseng-SDF	In vitro	Decreases DPPH, ABTs, and ferric ion radicals	[117]
(4)	WGAP, WGNP	Caenorhabditis elegans	Scavenges hydroxyl radicals and reduces ROS and lipid peroxidation	[31]
(5)	WGPN, WGPA	STZ-induced diabetic mice	Decreases MDA level and increases SOD activity	[35]
(6)	WGPA	Forced swim test/ICR mice	Inhibits MDA and LDH levels and causes the increases of SOD and GSH-Px activities	[118]
(7)	WGPA, WGPA-A	Forced swim test/ICR mice	Lowers MDA level and enhances GSH-Px activity	[59]
***American ginseng polysaccharides***				
(1)	AEP-2	RAW 264.7 macrophages	Shows higher values of Trolox equivalent and oxygen radical antioxidant capacities	[38]
***Notoginseng polysaccharides***				
(1)	FPNP	H_2_O_2_-induced human dermal fibroblast	Decreases ROS and MDA and increases the activities of CAT, GSH-Px, and SOD by activating the TGF-β/Smad signaling pathway	[2]
**Other bioactive functions**				
***Ginseng polysaccharides***				
(1)	GPS	C57BL/6 mice model	Promotes hypothalamic neuropeptide Y expression and inhibits the levels of proopiomelanocortin and dopamine D1 receptor in the midbrain to promote food intake	[119]
(2)	GPS	Ethanol-induced gastric injured rats	Inhibits oxidative stress (increased SOD and CAT and decreased MDA) and inflammation (reduced TNF-α, IL-6, IL-1β, and MPO) by inhibiting the NF-κB and STAT pathways	[27]
(3)	WGP	Antibiotic-associated diarrhea mice	Changes gut microbiota composition and diversity, balances metabolic processes to recover mucosal structure	[69]
(4)	WGP	Cisplatin-induced mice	Regulates the PI3K/AKT, PERK-eIF2α-ATF4, and NF-κB p65 signaling pathways to prevent endoplasmic reticulum stress, inflammatory response, and apoptotic cell death	[120]
(5)	GP	Diabetic rat model	Enhances Rb1 biotransformation by gut microbiota and promotes the fecal β-d-glucosidase activity	[121]
(6)	GP	Dextran sulphate sodium-induced colitis rat model	Enhances microbial deglycosylation and Rb1 intestinal absorption	[122]
(7)	APG	γ-rays irradiation-induced C57BL/6 mice model	Inhibits the p53-dependent and mitochondrial apoptosis pathways to protect small intestine	[123]
(8)	GP	H1N1 influenza virus infected BALB/c mice	Lowers lung viral titers and IL-6 to improve survival	[124]
(9)	AP1	Hypoxia/reoxygenation-induced H9c2 cells	Increases glucocorticoid receptor and estrogen receptor to activate the reperfusion injury salvage kinase pathway and regulates the eNOS-dependent pathways to maintain endothelial function	[125]
(10)	WGPA	ICR mice model	Increases social interactions and decreases aggressive behaviors	[65]
(11)	ELHPP-RGBPs	Dermatophagoides farinae extracts-induced NC/Nga mice	Inhibits the AP-1/MMP-1 pathway to prevent solar ultraviolet-induced skin wrinkles and atopic dermatitis	[126]
***Notoginseng polysaccharides***				
(1)	PNPS	Ischemia/reperfusion injured rat model	Regulates Bcl-2/Bax ratio and caspase-3 cascade to suppress apoptosis	[127]
(2)	PNPS-0.5M	Alcoholic liver damage mice model	Regulates the alcohol dehydrogenase and catalase pathways to prevent peroxide accumulation	[43]

**Abbreviations:** TNF-α, tumor necrosis factor-α; IL, interleukin; NO, nitric oxide; IFN-γ, interferon-γ; NK, natural killer; ConA, Concanavain A; LPS, lipopolysaccharides; BMP2, bone morphogenetic protein-2; CTX, cyclophosphamide; ERK, extracellular signal-regulated kinase; JNK, c-Jun N-terminal kinase; NF-κB, nuclear factor kappa B; AP-1, Activator protein-1; TLR4, Toll-like receptor 4; PI3K, Phosphatidylinositol 3-kinase; MCP-1, monocyte chemoattractant protein-1; GM-CSF, granulocyte-macrophage colony stimulating factor; DPPH, 1,1-diphenyl-2-picrylhydrazyl; ABTs, 2′-Azinobis-(3-ethylbenzthiazoline-6-sulphonate); ROS, reactive oxygen species; MDA, malondialdehyde; SOD, superoxide dismutase; LDH, Lactic dehydrogenase; GSH-Px, glutathione peroxidase; CAT, catalase; TGF-β, trans-forming growth factor-β; MPO, Myeloperoxidase; STAT, signal transducer and activator of transcription; AKT, Protein kinase B; eNOS, endothelial nitric oxide synthase; MMP, Matrix metalloproteinase; Bcl-2, B-cell lymphoma-2; Bax, BCL2-Associated.

## Data Availability

Not applicable.

## Abbreviations

AG, arabinogalactans; HG, homogalacturonan; RG, rhamnogalacturonan; EDTA, ethylene diamine tetraacetic acid; Gal, galactose; GalA, galacturonic acid; Ara, arabinose; Rha, rhamnose; TNF-α, tumor necrosis factor-α; IL, interleukin; NO, nitric oxide; IFN-γ, interferon-γ; CTX, cyclophosphamide; ERK, extracellular signal-regulated kinase; JNK, c-Jun N-terminal kinase; NF-κB, nuclear factor kappa B; LPS, lipopolysaccharides; MCP-1, monocyte chemoattractant protein-1; DPPH, 1,1-diphenyl-2-picrylhydrazyl; ROS, reactive oxygen species; MDA, malondialdehyde; CAT, catalase; GSH-Px, glutathione peroxidase; SOD, superoxide dismutase; TGF-β, trans-forming growth factor-β; GM-CSF, granulocyte-macrophage colony stimulating factor
